# Aligning deep learning and interpretable models for blood cell classification: A dual-model framework for explainability

**DOI:** 10.1371/journal.pdig.0001514

**Published:** 2026-07-30

**Authors:** Magdalene Yeok Yu Cheong, Xinran Xu, Li Rong Wang, Timothy Xiao Jing Yeo, Hemalatha Shanmugam, Shu Ping Lim, Bingwen Eugene Fan, Xiuyi Fan

**Affiliations:** 1 College of Computing and Data Science, Nanyang Technological University, Singapore, Singapore; 2 Lee Kong Chian School of Medicine, Nanyang Technological University, Singapore, Singapore; 3 A*STAR Centre for Frontier AI Research, Singapore, Singapore; 4 Department of Haematology, Tan Tock Seng Hospital, Singapore, Singapore; 5 Department of Laboratory Medicine, Tan Tock Seng Hospital, Singapore, Singapore; 6 Department of Laboratory Medicine, Khoo Teck Puat Hospital, Singapore, Singapore; 7 Yong Loo Lin School of Medicine, National University of Singapore, Singapore, Singapore; 8 Centre for Medical Technologies & Innovations, National Health Group, Singapore, Singapore; Oxford University: University of Oxford, UNITED KINGDOM OF GREAT BRITAIN AND NORTHERN IRELAND

## Abstract

Blood smear examination involves classifying cells by morphology under a microscope, a labour-intensive process prone to subjective variation. Recent deep learning models achieve strong performance in blood cell classification but remain as “black boxes”, offering little clinical transparency. We propose a dual-model framework that pairs a deep YOLO (You Only Look Once) classifier with a shallow, interpretable explainer. YOLO performs classification and segmentation, while clinically informed features are extracted from segmented images to train the explainer on YOLO predictions. SHapley Additive exPlanations (SHAP) quantify feature importance, with discrepancies flagged as “other reasons” to enhance transparency. Evaluated on proprietary and public datasets (PBC, Raabin), YOLO achieved AUCs of 0.997, 0.994, and 1.000 respectively, with the explainer demonstrating strong alignment: Score-CAM activation maps agreed with SHAP-identified features. In a user study with 9 trained and qualified haematologists and laboratory medical technologists, predictions achieved 96.9% concurrence. This work shows that the coupling of deep learning with interpretable models improves the confidence of predictions while providing clinical meaningful insights.

## Author summary

Interpretable machine learning for medical diagnostics is essential for ensuring trust and usability in medical decision-making systems. However, many high-performance deep learning models lack transparency, making it difficult for clinicians to understand their predictions. For blood cell classification, existing explainable AI (XAI) techniques are not able to relate predictions to morphological properties of blood cells. This limits the application of deep learning models for medical diagnostic applications. In this study, we first used a deep learning model, YOLO, to classify the cell name. Next, we segment the cell and create engineered features like size, circularity, number of nuclei lobes, for example. Our explanation model predicts the cell name using these engineered features. YOLO demonstrates good classification performance against hand-labelled ground truths while the explanation model achieves good classification performance against YOLO predictions. The study also shows that Score-CAM on YOLO predictions and SHAP analysis of the explainer’s predictions highlight consistent regions, demonstrating alignment between the deep learning model and the interpretable explainer. The strong performance of the Random Forest explainer further indicates tight coupling with YOLO while providing explicit reasons for the predictions in terms of morphological properties.

## Introduction

Haematology is the branch of medicine that revolves around the study and diagnosis of diseases and disorders related to blood. Blood morphology provides critical diagnostic information on cell abnormalities indicative of diseases or disorders, like anaemia and leukaemia [1,2]. A laboratory haematologic diagnosis consists of a complete blood count and blood smear examination. In routine hospital practice, initial screening is typically performed using automated haematologic analysers; however, these systems cannot fully replace manual blood smear examination. A previous study reported that 26.7% of specimens still required verification by manual smear review [3]. Manual blood smear examination involves visual inspection of red blood cells (RBCs), white blood cells (WBCs), and platelets under a microscope and classification according to their morphology (e.g., size, shape, colour, and granularity). This process is time-consuming, requiring on average about 30 minutes per specimen [4], and is subject to human bias and skill differences, resulting in documented inter-observer variability in examination results [5].

Research work on employing machine learning for cell classification has long garnered strong interest. In these works, researchers extract cell morphological properties and leverage on the differences in these properties to classify cells. In one such early work, Street et al. [6] demarcated boundaries of cell nuclei and extracted features such as perimeter, area, compactness and classified the cells based on these extracted features using Multi-surface Method to define separating planes. However, this method does not work well for large datasets. It is also not able to separate non-linear, non-binary or complex datasets with feature interactions.

Subsequently, other classical machine learning methods like Support Vector Machine (SVM), Decision Tree (DT) and K-Nearest Neighbour (KNN), to name a few, were evaluated to circumvent these constraints and difficulties [7]. These methods are able to classify multiple classes and are more adaptable to different datasets. Methods like SVM overcome difficulties in separating non-linear datasets using kernels. A similar study by Lamberti [8] used SVM with a polynomial kernel to classify RBCs, WBCs and platelets using shape-based features, such as eccentricity, extracted from segmented cells. However, these methods rely on engineered features where spatial information is lost and may miss information on fine-grained features with subtle differences between cell types. Deep learning models, on the other hand, are designed to process images at the pixel level, capturing both spatial and hierarchical relationships. They are also capable of modelling complex data structures and learning non-linear patterns that traditional methods may struggle to represent [9].

The advent of deep learning has significantly advanced blood cell classification, offering automated solutions with high accuracy. Convolutional Neural Networks (CNNs) have proven to classify blood cells with high performance metrics based on a recent survey [10]. However, they repute as “black boxes”; how they arrive at their predictions cannot be fully understood. The lack of transparency in deep learning models has led to substantial hesitation in adopting them for medical applications. To address this impediment, Explainable Artificial Intelligence (XAI) techniques have been explored to provide interpretable justifications for model predictions [11]. For example, in a survey with clinicians, respondents reported that trust in an AI system depends not only on its accuracy, but also on whether its explanations accord with clinical intuition during time-critical decision-making [12]. Furthermore, studies in high-risk medical environments have shown that integrating XAI methods—such as visual saliency maps or concise textual rationales—can increase user trust, reduce perceived risk, and improve willingness to rely on AI-generated decisions [13]. Such transparency is essential for the medical community to fully embrace AI for diagnostic applications.

There are several established XAI techniques. Methods such as Gradient-weighted Class Activation Mapping (Grad-CAM) overlay heatmaps on input images to highlight regions that influence the model’s predictions [14]. While these visual explanations indicate areas of interest, they do not quantify morphological features in terms of cell *shapes* and *structures* to explain the prediction. This discrepancy underscores the need for explanations that are both faithful to the model’s workings and clinically meaningful [15]. Therefore, the challenge for AI practitioners is to empower a strong classifier with convincing explanations in a self-contained scalable framework.

To accomplish this, we develop the **Dual-Model REasoning for Automated Morphology (DREAM)** framework, a dual-model architecture that combines the strengths of deep learning and interpretable machine learning. DREAM utilises the You Only Look Once (YOLO) model [16] for precise blood cell detection and classification, followed by segmentation using K-Means Clustering and the Segment Anything Model (SAM2) [17,18] to isolate cellular components. From these segmented images, we extract engineered features that capture clinically relevant morphological characteristics. These features are then used to train a Random Forest (RF) model, which is optimized to mirror YOLO’s classification outputs, ensuring alignment between the two models. Finally, we apply SHapley Additive exPlanations (SHAP) [19] to the RF model to quantify the importance of each feature, providing transparent and clinically interpretable explanations for the classifications.

Our experimental results demonstrate that DREAM achieves good classification performance. DREAM is evaluated on 3 datasets: proprietary and 2 public datasets (PBC and Raabin). The prediction model, YOLO, achieves average precision and recall across classes against ground truth at 100% and 99.7% on proprietary dataset, 99.6% and 99.8% on PBC public dataset, and 100% and 100% on Raabin public dataset. In turn, the explainer (RF) achieves average precision and recall across classes against YOLO prediction at 99.0% and 98.5% on proprietary dataset, 99.4% and 99.0% on PBC public dataset, and 97.4% and 97.6% on Raabin public dataset. Score-CAM activation maps for the YOLO classifier match with RF explainer top features identified by SHAP analysis. Additionally, the RF explainer, with the engineered features, trained against ground truth, achieves average precision and recall across classes at 99.2% and 98.9%. This demonstrates that the extracted morphological features contribute to transparent decision-making: they are representative of the ground truth, and YOLO predictions.

A user study with 9 trained and qualified haematologists and laboratory medical technologists confirms that the explanations align well with expert assessments (96.9%), reinforcing the clinical relevance and interpretability of the proposed framework.

The three key contributions of this work are as follows:

1. **Novel Dual-Model Framework**: Integration of YOLO for classification and Random Forest for explanation in terms of cell morphological properties, delivering accuracy and interpretability.
2. **Clinically Relevant Feature Extraction**: Built-in automatic generation of morphological features from segmented images.
3. **Enhanced Model Transparency**: Utilization of SHAP to provide detailed feature importance, aligning model predictions and clinical understanding.

## Related work

### Blood cell classification

Numerous studies have highlighted the efficacy of deep learning models in white blood cell (WBC) classification. Before the era of deep learning, blood cell classification is evaluated using extracted features with classical machine learning algorithms. Elen et al. [7] evaluated 6 classical machine learning algorithms to classify cells using 35 geometric and statistical features. Examples of geometric features included radius, area and number of lobes. Examples of statistical (texture) features included mean, standard deviation and kurtosis. The accuracy achieved was: 81.61% for Decision Tree (DT) and K-Nearest Neighbour (KNN), 82.76% for Naive Bayes (NB), 84.48% for Support Vector Machine (SVM), 91.38% for RF and 96.55% for Logistic Regression. In comparison to an earlier work, Gautam et al. [20] extracted geometric features from the nucleus, including area, perimeter, circularity, and eccentricity, and achieved 80.88% accuracy with a Naive Bayes classifier.

In another work, Dasariraju et al. [21] generated 16 features based on nucleus size, nucleus shape, elliptical features and colour features, and trained with Random Forest (RF), achieving precision from 69.23% to 100% and recall from 75% to 100%. This study was conducted on *erythroblast, monoblast, promyelocyte* and *myeloblast*.

Neural networks are also evaluated for WBC classification using extracted features. Su et al. [22] first segmented the cellular components of WBCs and extracted geometric, colour, and textural features from these regions. These hand-crafted features were then used as inputs to train three classifiers: a support vector machine (SVM), a hyperrectangular composite neural network (HRCNN), and a multilayer perceptron (MLP). SVM achieved an accuracy of 92.72%, HRCNN 66.90% and MLP 98.01%. Manik et al. [23] and Hegde et al. [24] subsequently used artificial neural networks (ANNs) to classify cells based on extracted features, achieving accuracies of 98.90% and 99.00% respectively. All 3 works reported much higher accuracy for neural networks than classical machine learning methods.

Subsequently, deep learning models (CNNs) are evaluated for blood cell classification. In deep learning approaches, both feature extraction and classification are learned jointly within the network. Commonly used convolutional architectures include VGG and ResNet variants. For example, Asghar et al. [24] employed transfer learning with pre-trained CNNs for classifying basophils, eosinophils, lymphocytes, monocytes and neutrophils, reporting average accuracies of 96.86% for VGG16, 92.40% for VGG19, 95.56% for ResNet50, 91.60% for ResNet101, and 96.00% for ResNet152. In their setting, the models were trained and evaluated on full-field images containing both WBCs and background. Such configurations can be sensitive to variations in image composition; for instance, performance may degrade when a test image contains multiple WBCs or substantially different red blood cell (RBC) density as compared to the training images, potentially challenging generalisation in real-world smear examinations.

Some works, however, do not apply deep learning model directly. Elhassan et.al. [25] proposed a two-stage hybrid model. The first stage combined a geometric transformation (GT) and a deep convolutional autoencoder (DCAE) to develop the GT-DCAE model for augmentation and generation of feature maps, which were then used in Convolutional Network (CNN) for classification. In the first stage, the model classified normal from abnormal cells. Second stage classification was done only on abnormal cells.

On the whole, it is observed that deep learning models demonstrate stronger classification performance over classical machine learning models. Table 1 shows the performance of WBC classification with classical machine learning and various deep learning models. While classical machine learning models (linear models, decision trees) often permit relatively direct inspection of their parameters or decision rules, explaining the predictions of deep learning models remains challenging due to their high-dimensional, deeply layered, and non-linear architectures, in which intermediate representations do not correspond to human-interpretable concepts in a straightforward manner [36]. In the medical domain, this ‘black-box’ nature has shown to hinder traceability and the rigorous validation required for clinical safety and regulatory acceptance, contributing to clinicians’ reluctance to rely on such systems in practice [11].

**Table 1:** Comparison of classification performance of related work. Cell types in this table are basophil, eosinophil, lymphocyte, monocyte and neutrophil unless stated otherwise.

Author	Year	Method	Data Source	Accuracy (%)	Remarks
Gautam et al. [20]	2016	Naive Bayes	–	80.88	extracted features on nucleus only
Elen et al. [7]	2019	Decision Tree	–	81.61	extracted geometrical and statistical
		K-Nearest Neighbour	–	81.61	features
		Naive Bayes	–	82.76	
		Random Forest	–	91.38	
		Support Vector Machine	–	84.48	
		Logistic Regression	–	96.55	
Su MC et al. [22]	2014	SVM	–	92.72	base on extracted geometrical,
		HRCNN	–	66.90	colour, and texture features.
		MLP	–	99.11	
Manik et al. [23]	2016	ANN	–	98.90	base on extracted geometrical
					and texture features.
					(eosinophil, lymphocyte, neutrophil)
Hegde et al. [26]	2019	ANN	–	99.00	base on extracted geometrical
					and texture features.
Almezhghwi et al. [27]	2020	ResNet-50	LISC	97.40	data augmentation with GAN
		DenseNet-121	LISC	98.30	data augmentation with GAN
		DenseNet-169	LISC	98.80	data augmentation with GAN
Girdhar et al. [28]	2022	CNN	–	98.55	–
Jiang al. [29]	2022	DRFA-Net	–	93.2 - 95.87	BCCD, LISC and Raabin datasets
Baghel et al. [30]	2022	CNN	BCCD	98.91	excluded basophil
Cheuque et al. [31]	2022	Multilevel CNN	–	98.36	–
Zhao et al. [32]	2022	YOLO5	BCCD	97.92	–
Katar et al. [33]	2023	ViT	Raabin	99.40	–
Zou et al. [34]	2025	AlexNet	–	96.96	combination of 4 public datasets
		VGG16	–	98.32	
		ResNet34	–	98.36	
		ResNet50	–	98.24	
		GhostNetv1	–	97.52	
		GhostNetv2	–	98.47	
		MobileNetv2	–	97.74	
		ICAFF-MobileNetv2	–	98.54	
Sherpa et al. [35]	2025	VGG16 (with SpinalNet)	Raabin	97.65 (98.32)	–
		VGG19 (with SpinalNet)	Raabin	93.02 (98.13)	
		ResNet50 (with SpinalNet)	Raabin	97.35 (98.36)	
		ResNet101 (with SpinalNet)	Raabin	98.13 (98.43)	
		DenseNet169	Raabin	97.53	
		EfficientNetB0	Raabin	97.40	
		Xception	Raabin	98.18	
		MobileNet	Raabin	97.35	
		MobileNetv2	Raabin	97.03	
		SqueezeNet	Raabin	89.51	

### Explainable AI

The need to explain model predictions has led to the development of several XAI techniques. Panthakkan et al. [37] proposed a deep learning ensemble model for WBC classification and used Grad-CAM to provide visual explanations by highlighting the image regions that influenced the model’s predictions. Similarly, Chen et al. [38] incorporated Grad-CAM into their proposed CNN architecture, which was built on ResNet and DenseNet backbones and integrated with spatial and channel attention modules, while Katar et al. [33] used Score-CAM to explain predictions from a Vision Transformer model. In their work [15], N. Arun et al. evaluated the trustworthiness of Grad-CAM and other gradient-based XAI methods on medical imaging applications, and reported sub-optimal reliability.

The above techniques use a Class Activation Map (CAM) to highlight the regions of the input image that most influence the model predictions. CAMs are heatmaps that encode the influence of different image regions on the model’s prediction through colour intensity. For example, a commonly used colour scheme maps regions of highest influence to bright red, with intensity gradually decreasing through yellow and green and finally to deep blue for regions of minimal influence.

There are many other techniques that engage CAMs, such as Layer-CAM [39] and Ablation-CAM [40]. However, all these techniques are not able to infer the morphological properties responsible for the predictions.

In another work, Kim et al. [41] made use of Concept Activation Vectors to classify based on image patterns. Similarly, this technique is also unable to identify the morphological properties responsible for the classifications.

Dhibar et al. [42] and Bhatia et al. [43] both used Local Interpretable Model-Agnostic Explanations (LIME) [44] in their respective WBC classification studies. Islam et al. [45] combined multiple XAI techniques, including Grad-CAM, LIME, and SHAP, to enhance the interpretability of their proposed CNN model for WBC classification. The above XAI methods, however, are not able to relate the predictions to morphological properties responsible for the predictions.

To relate deep learning model predictions to morphological properties, Michalski et al. [46] used SHAP DeepExplainer. The work reported on the percentage of overlap between SHAP DeepExplainer output image and separately segmented cellular components in terms of cytoplasm area, cell shape, nucleus area and nucleus shape. While it is able to attribute predictions to shapes when the overlap is high, it does not relate to the shape peculiar to each unique cell type - round or irregular - as numerical value like circularity is not available. It is also not able to relate predictions to morphological properties like granularity or number of lobes. Since a combination of these morphological properties is essential for differentiating the WBCs, the lack of discrete numerical values for comparison limits the overall explainability power and makes it challenging to scale to additional cell types.

In their study, S. Tsutsui et al. [47] created datasets by annotating a few cell types based on morphological properties. Examples of annotated features are if nuclear-cytoplasmic ratio is high or low and if granularity exists. The datasets were then used to train models to classify the cells. Pal et al. [48] built a transformer encoder-decoder based model to classify, segment and explain. However, significant effort was required to generate segmentation masks. Each sample was then annotated with categorical attributes, for example, if the cell was granular, if it was basophilic or eosinophilic, if the nucleus was bilobed or multilobed or was round or oval. However, extension to other cell types requires the same labour-intensive labelling and is not scalable for both methods. Additionally, the explainability power is also restricted. The methods do not provide numerical measurements to differentiate cytoplasm of different granule size, number of nuclei lobes or quantify circularity to inform the extent of roundness or irregularity, to name a few.

Although existing approaches have made significant strides in explainability, opportunities remain for approaches that provide clinically meaningful interpretations.

## Materials and methods

### Datasets

Our proprietary dataset contains a total of 23,227 360-by-360 pixels microscopic images prepared under standard haematological staining protocols (Wright’s stain). The peripheral blood smears are digitised using a CellaVision DI-60 analyser, which automatically detects WBC candidates and exports cropped single-cell images, typically within one minute per slide. DREAM operates on these pre-cropped images as a downstream software module, so no additional manual cropping is required for prospective use. Each image is manually annotated by expert haematologists to ensure precise labelling and serves as the ground truth for classification. We also evaluated the framework on 2 public datasets, PBC [49] and Raabin [50]. There are differences between the datasets in terms of staining methods, image size and cell types. Table 2 lists the details of the 3 datasets.

**Table 2:** Dataset details.

	Proprietary	PBC	Raabin
Number of images	23,227	9,730	2,670
Image size	360-by-360	360-by-363	575-by-575
Staining	Wright’s stain	Grünwald-Giemsa	Giemsa
Labelling	minimum 2 experts	2 experts	mostly 2 experts
**Cell Type:**			
Basophil	Yes	Yes	Yes
Eosinophil	Yes	Yes	Yes
Large Granular Lymphocyte	Yes	No	No
Lymphocyte	Yes	Yes	Yes
Monocyte	Yes	Yes	Yes
Segmented Neutrophil	Yes	Yes	Yes

**Fig 1:**
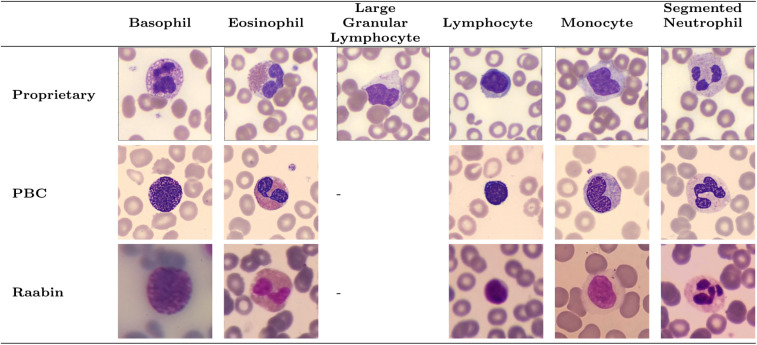
Cell types used in proprietary and public datasets.

Each of the selected cells has a distinguished set of characteristics. For example, *lymphocyte* has small cell size and high nuclear-cytoplasmic ratio while *monocyte* is much larger and has lower nuclear-cytoplasmic ratio. While both *eosinophil* and *segmented neutrophil* could be multi-lobed, they differ in cytoplasm colour and granularity. Fig 1 shows examples of the cells used in the datasets. We observe huge differences in cell appearance for different datasets. Distinction between nucleus and cytoplasm is low for *basophil* in the PBC dataset while *basophil* in the Raabin dataset is blur. *Eosinophil* and *monocyte* appear unusually redder in the Raabin dataset.

The segmentation module is evaluated on Mendeley public dataset [51]. The dataset consists of 400 120-by-120 pixels microscopic images of unlabelled WBC with corresponding nucleus and cytoplasm masks. The segment masks serve as ground truths to measure against the performance of our segmentation module.

### Methodology

**Fig 2:**
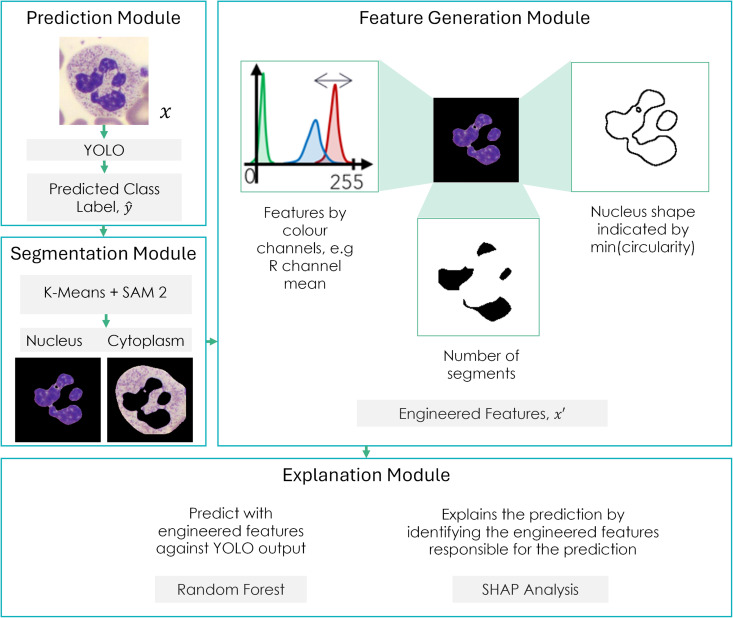
The DREAM framework.

As illustrated in Fig 2, the DREAM framework consists of four components:

1. **Prediction Module**: The first component detects and classifies the cell with YOLO object detection and classification model.
2. **Segmentation Module**: The second component generates the nucleus and cytoplasm masks, which are used to crop out the segments in the input.
3. **Feature Generation Module**: Clinically relevant quantitative values like colour channel intensity and circularity are generated from the cropped segments, forming the engineered features.
4. **Explanation Module**: Engineered features are used to train a random forest model (RF), with SHAP explanations produced for its predictions.

### Prediction module

The prediction module is implemented with YOLO11s, a single-stage detector that directly predicts bounding boxes and class probabilities from the full image in a single forward pass [16]. Compared with two-stage detectors, this design offers substantially faster inference, which is important for time-critical, high-throughput clinical laboratory workflows [52]. YOLO’s multi-scale feature maps also capture contextual information around each cell, improving robustness against misclassifying background artefacts as WBCs and allowing multiple WBCs in the same field of view to be detected and classified simultaneously. The suitability of YOLO for medical imaging has been demonstrated in several applications, including real-time WBC detection and malaria parasite detection [53,54], supporting its use as the backbone prediction network in DREAM.

We compared YOLO8 and YOLO11 across different batch sizes (32, 64, and 128) and different model sizes (“s” & “m”). Performance is similar for different models, model variants and batch sizes. YOLO11s (batch size 128) is selected for its efficiency. The bounding box result from the prediction is used to crop out the detected WBC in preparation for segmentation. This helps to reduce background noises from interfering with segmentation performance.

### Segmentation module

**Fig 3:**
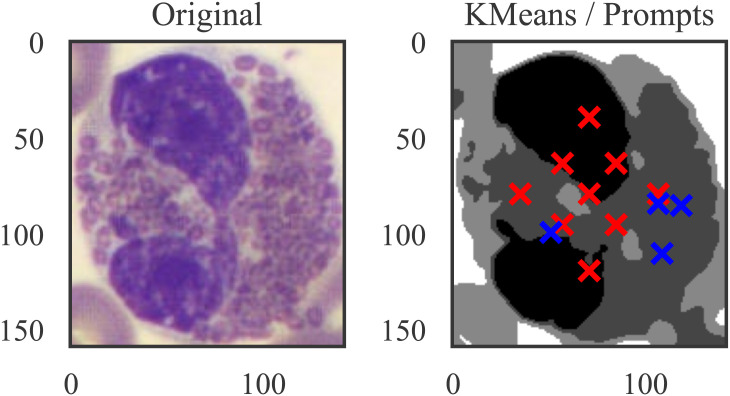
SAM 2 prompt positions of a Eosinophil. Red ‘X’ represents fixed positions. As the detected cell is centered within the bounding box, the midpoint (midpoint of width and height) is certainly correctly landed within the cell. The remaining point prompts are confined to within 25% of the bounding box width and height from the centre. The fixed point prompts, automatically customised for each image, are thus designed to situate within 50% of the bounding box width and height in the middle of the bounding box to ensure correct landing. Blue ‘X’ represents randomly selected positions within the cytoplasm.

The *Segmentation* module is a combination of K-Means clustering and SAM 2. Clustering helps to differentiate nucleus and cytoplasm from red blood cells and background. We make use of clustering to obtain a coarse segmentation of the nucleus and refine the nucleus mask through dilation and blurring. We extract the index positions of the nucleus and cytoplasm from the clustering results, which serve as prompts to instruct SAM 2. The number and placement of prompts are optimized through experiments. For SAM2-based segmentation, we use 13 point prompts per detected cell. Nine fixed prompts are placed within the YOLO bounding box so that, given the centered cell, they reliably fall inside the cell and provide consistent coverage. Four additional random prompts are sampled from pixels in the cytoplasm cluster identified by K-means to refine cytoplasmic boundaries. Fig 3 illustrates these fixed (red ‘X’) and random (blue ‘X’) prompt positions for an eosinophil as an example. Once extracted, the cell mask undergoes refinement through dilation, blurring, and mask subtraction to improve clarity and eliminate noise. This method eliminates the need for mask annotation data for model training and inference.

### Feature generation module

The *Feature Generation* module computes quantitative descriptors that capture the clinical characteristics of each cell type. Following the feature design in [55], we defined 18 engineered features, as summarised in Table 3. With the exception of circularity, all features are computed directly from the nucleus and cytoplasm segments. For example, nuclear and cytoplasmic sizes are obtained by computing the pixel area of each segment using the contour-area function in the OpenCV library, and total cell area is defined as the sum of these two components. Chromatin pattern and cytoplasmic granularity are quantified by the standard deviation of colour intensity within the nucleus and cytoplasm segments respectively; this statistic serves as a proxy for textural heterogeneity, distinguishing, for instance, the highly granular cytoplasm of basophils from the more uniform cytoplasm of monocytes. Circularity is derived from the area and perimeter of each segment using Eqn 1. In this work, the terms “cell shape” and “nucleus shape” refer to circularity, where value of 1 corresponds to a perfect circle.


$$\begin{array}{c} \text{Circularity}=4\times \pi \times \frac{\text{area}}{{\text{perimeter}}^{2}} \end{array}$$ (1)

**Table 3:** Cell characteristics and the corresponding engineered clinical features. Features labelled with (^*^) are retained after feature selection.

Cell Region	Clinical Characteristics	Engineered Features	Feature Count
Whole	Size	YOLO Bounding Box	1
		Area of Cell^*^	1
	Shape	Circularity of Cell^*^	1
	Nuclear-cytoplasmic Ratio	Nucleus Size / Cytoplasm Size^*^	1
Nucleus	Size	Area of Nucleus	1
	Shape	Circularity of Nucleus^*^	1
	Number of Lobes	Number of Nuclei Lobes ^*^	1
	colour	Means of RGB Channels^*^	3
	Chromatin Pattern	Standard Deviation of RGB Channels^*^	3
Cytoplasm	Size	Area of Cytoplasm	1
	colour	Means of RGB Channels^*^	3
	Granularity	Standard Deviation of RGB Channels^*^	3

Although Eqn 1 does not directly measure shape, it indicates how circular the shape is. This helps to differentiate round cells like *lymphocyte* from irregularly-shaped cells like *large granular lymphocyte*. For an accurate count of nuclei lobes, we first dilate the nucleus mask to separate lobes that are connected by filaments before counting. Features are also derived from each colour channel. An additional cell size feature is derived from YOLO detection bounding box output.

### Explanation module

The *Explanation* module consists of a Random Forest classifier (150 trees, maximum depth 15) trained on the engineered morphological features, together with SHAP analysis for explanation generation. SHAP explains predictions by quantifying the importance of each feature. The RF classifier is trained to predict output of the *Prediction Module*. To ensure that the explanations provided by the RF model aligns well with the YOLO classifier, we explicitly account for discrepancies between the two models.

Let $f_{o}$ be the YOLO classification model where $f_{o}(𝐱)\in [0,1]$ represent the class probability assigned to the prediction class *c* by the classifier for an image **x**, and let $f_{r}(g(𝐱))\in [0,1]$ be the corresponding probability for class *c* computed by the RF, where the feature engineering function g(𝐱) maps images to *n* dimension vectors. The discrepancy ϵ between the two models is:


$$\begin{array}{c} \epsilon =f_{o}(𝐱)-f_{r}(g(𝐱)). \end{array}$$ (2)

To provide a comprehensive explanation on the outcome of the classification model, we extend the standard SHAP formulation to explicitly incorporate ϵ.

With the standard SHAP applied to $f_{r}$, a prediction is explained as:


$$\begin{array}{c} f_{r}(g(𝐱))=\phi_{0}^{\left(e\right)}+\sum_{i=1}^{n}\phi_{i}^{\left(e\right)}, \end{array}$$ (3)

where $\phi_{0}^{\left(e\right)}$ is the baseline prediction for $f_{r}$ and $\phi_{i}^{\left(e\right)}$ is the contribution of feature $g_{i}(𝐱)$. To explain $f_{o}(𝐱)$, we set


$$\begin{array}{c} f_{o}(𝐱)=\phi_{0}^{\left(e\right)}+\sum_{i=1}^{n}\phi_{i}^{\left(e\right)}+\phi_{\epsilon }, \end{array}$$ (4)

where $\phi_{\epsilon }$ is the “contribution” of ϵ to $f_{o}(𝐱)$. From Eqns 2 and 3, we have $\phi_{\epsilon }=\epsilon$. Thus, ϵ is entirely attributed to itself, in a two-feature game with one feature being the output of the RF model, while the other feature being the residual ϵ. Thus, for an image **x** with its YOLO prediction $f_{o}(𝐱)$, the *explanation* is:


$$\begin{array}{c} \langle \phi_{1}^{\left(e\right)},\ldots ,\phi_{n}^{\left(e\right)},\phi_{\epsilon }\rangle . \end{array}$$ (5)

Explicitly incorporating $\phi_{\epsilon }$ ensures faithful explanation to YOLO’s prediction.

## Results

### Classification model

The experiment is first evaluated on the proprietary dataset and then validated on 2 public datasets. The results shown below are for the proprietary dataset unless stated otherwise. The proprietary dataset is imbalanced. Data augmentation is carried out for *basophil, large granular lymphocyte* and *lymphocyte* by random selection of 8 permutations of horizontal and vertical flips, rotations and colour balance variations. However, data augmentation did not improve performance. It is likely that the model is invariant to the applied transformations, which have not introduced sufficient semantic variability to improve training. YOLO11 precision and recall are 0.98 and 0.99 without augmentation, and 0.97 and 0.99 with augmentation respectively.

**Fig 4:**
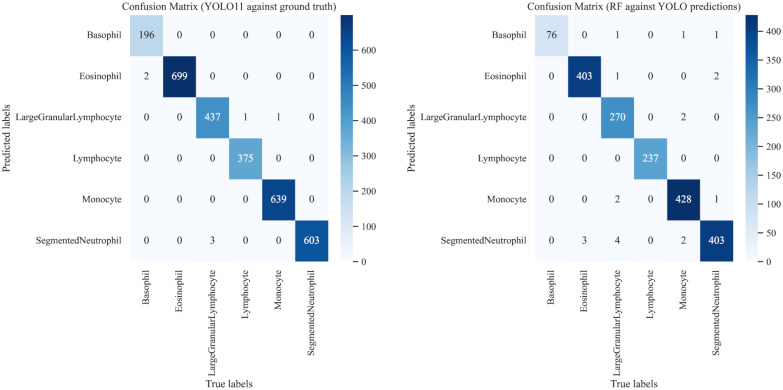
Confusion matrices of YOLO11 against ground truth and RF against YOLO11.

The proprietary dataset is split into training, validation, and test sets in a 70:20:10 ratio to ensure robust evaluation. YOLO8 and YOLO11 are evaluated for classification, with their results shown in Table 4. The high classification results are aligned with state-of-the-art works, such as the one shown in [28]. Performance between YOLO8 and YOLO11 is comparable. YOLO11, with approximately 75% FLOPs of YOLO8, is chosen for its efficiency. Results to be shown together under Explanation model section. Fig 4 shows the confusion matrices for YOLO and for the RF classifier on the test set. These matrices allow inspection of per-class performance and error patterns, and illustrate that the RF surrogate closely follows YOLO’s predictions while providing an interpretable feature-based rationale.

**Table 4:** Classification results of prediction and explanation models for propriety and public datasets. The two YOLO models, Y8 and Y11, are evaluated against the ground truth (GT), which consists of human labels. The RF model is assessed both against a classification model (RF-Y11) and the ground truth (RF-GT). All models exhibit high classification accuracy, with the explanation model being well aligned with the classification model (RF-Y11). The reported results represent mean values over 10 runs, with a standard deviation of less than 0.01 throughout. DREAM framework is further evaluated on 2 public datasets show strong performance. Number of RF-Y11 mispredictions on Raabin is up to 2.

	Basophil	Eosinophil	Large Granular	Lymphocyte	Monocyte	Segmented
			**Lymphocyte**			**Neutrophil**
**Proprietary dataset**						
Num. of Samples	1054	4953	3428	3133	5179	5480
Precision (Y8-GT) Mean	1	0.9971	0.9932	1	1	0.9967
Recall(Y8-GT) Mean	0.9899	1	0.9977	0.9974	0.9969	0.9983
Precision (Y11-GT) Mean	1	0.9971	0.9954	1	1	0.9950
Recall(Y11-GT) Mean	0.9899	1	0.9932	0.9973	0.9984	1
Precision (RF-GT) Mean	1	0.9951	0.9819	1	0.9884	0.9854
Recall (RF-GT) Mean	0.9744	0.9902	0.9891	1	0.9907	0.9878
Precision (RF-Y11) Mean	1	0.9926	0.9712	1	0.9885	0.9902
Recall(RF-Y11) Mean	0.9620	0.9926	0.9926	1	0.9930	0.9782
**PBC dataset**						
Num. of Samples	1073	2933	–	1161	1358	3205
Precision (Y11-GT) Mean	0.9874	1	–	0.9858	1	1
Recall(Y11-GT) Mean	1	1	–	1	0.9878	0.9984
Precision (RF-Y11) Mean	0.9867	0.9954	–	1	0.9894	0.9872
Recall(RF-Y11) Mean	0.9737	0.9954	–	0.9881	0.9894	0.9957
**Raabin dataset**						
Num. of Samples	295	737	–	498	547	593
Precision (Y11-GT) Mean	1	1	–	1	1	1
Recall(Y11-GT) Mean	1	1	–	1	1	1
Precision (RF-Y11) Mean	0.9583	0.9608	–	1	1	0.9524
Recall(RF-Y11) Mean	0.9583	0.9608	–	1	0.9762	0.9756

### Segmentation and feature generation

**Fig 5:**
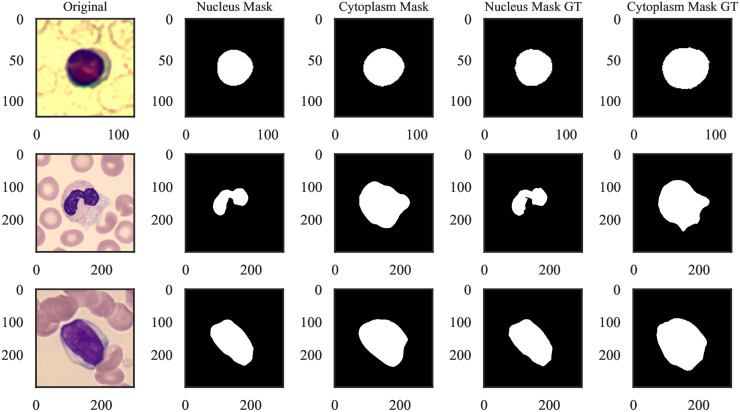
Segmented nucleus and cytoplasm and the corresponding ground truths. General appearance of the segmented parts are similar to ground truth (GT), with more differences observed on the cytoplasm. Segmentation on the cytoplasm appears to be challenging for cells with pale cytoplasm which makes it harder for SAM 2 to differentiate against the background.

The segmentation module was quantitatively evaluated on the Mendeley public dataset [51], which includes expert-annotated nucleus and cytoplasm masks. Our KMeans–SAM2 pipeline was applied to each cell, and the predicted nucleus and cytoplasm segments were compared against the corresponding ground truths using IoU and Dice coefficients. Table 5 shows IoU and DICE results. Intersection of segments produced by our segmentation module and the ground truths achieves IoU above 85% and DICE above 91%. Representative examples are shown in Fig 5. While the overall agreement is high, cytoplasm segmentation is slightly less accurate, particularly for cells with pale cytoplasm that are harder to distinguish from the background.

**Table 5:** IoU and DICE results for segmentation evaluation on Mendeley public dataset.

	Nucleus Segment	Cytoplasm Segment
IoU Mean	0.8819	0.8594
IoU SD	0.0931	0.1472
DICE Mean	0.9342	0.9147
DICE SD	0.0621	0.1257

**Fig 6:**
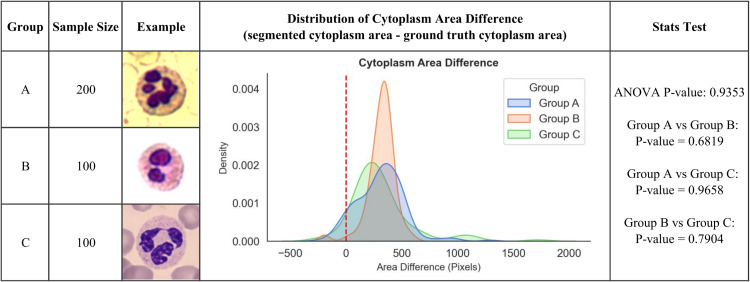
Segmentation evaluation results on the cytoplasm. Groups A, B and C showed distinct colour characteristics. All exhibited slight cytoplasm over-segmentation. ANOVA showed no statistically significant difference among groups.

The challenge in cell segmentation lies primarily in detecting the interface between cytoplasm and background. The Mendeley dataset consists of three image groups with distinct colour characteristics. We therefore compared segmentation performance across Groups A, B, and C by analysing the area difference between the segmented cytoplasm and the corresponding ground-truth cytoplasm mask. Fig 6 shows representative examples and the distribution of cytoplasm area differences. All three groups showed slight over-segmentation, and ANOVA indicated no significant difference among them. These results suggest that the segmentation method is reasonably robust to colour variation due to staining and imaging conditions. As ground-truth segmentation masks are unavailable for the proprietary dataset, IoU/Dice-based evaluation is performed only on the Mendeley dataset. To ensure reliable feature extraction, for training the explanation model on the proprietary dataset, we instead apply a post-segmentation plausibility filter by retaining only cells whose computed cell area falls within a cell-type-specific mean ± 1 standard deviation range.

**Fig 7:**
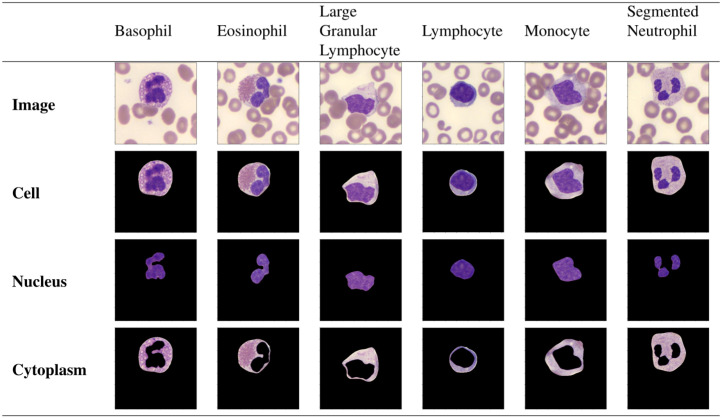
Cell types and the segmented parts in proprietary dataset.

**Fig 8:**
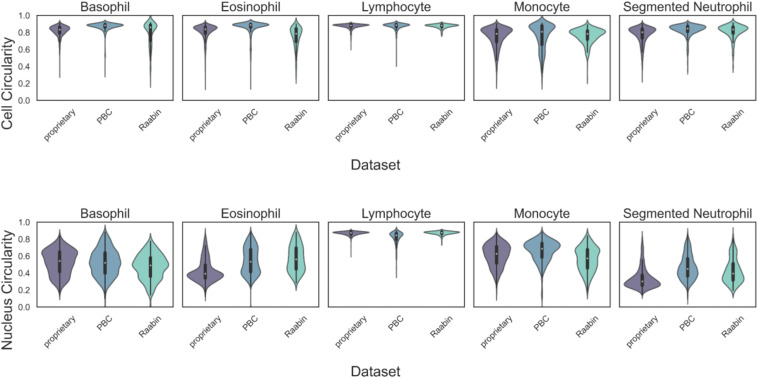
Examples of values of engineered features for proprietary and public datasets. *Large Granular Lymphocyte* is not shown as both public datasets do not have this cell type.

Fig 7 illustrates the 6 cell types and the subsequent segmented parts. Fig 8 shows the examples of values of the engineered features between the proprietary dataset and the public datasets. With reference to plots for *Lymphocyte*, both cell circularity and nucleus circularity are higher than the other cells. This reflects morphological properties of lymphocytes - round nucleus and round cell. This demonstrates that the Feature Extraction Module is able to extract from the segmented parts information that reflects the morphological properties. The full set of data for all engineered features are shown in the Supporting information section (S1–S3 Figs).

### Explanation model

Feature selection for the explanation model was conducted using a combination of correlation analysis and clinical knowledge. Highly correlated features providing redundant information were excluded. For example, cytoplasm area was omitted as it was directly derived from the difference between the cell and nucleus areas. However, despite correlations among the RGB channels, they were retained, as each channel contributes distinct qualitative information to the image. Removing them would compromise interpretability by limiting the explanatory power of the model. The final selected features are those labelled with (*) in Table 3.

On the proprietary dataset, YOLO correctly classifies 99.84% of ground truth labels. With the selected features, RF correctly predicts 98.91% of YOLO classifications. Thus, both models are accurate and “confident” about their predictions.

DREAM is further validated on 2 public datasets, PBC [49] and Raabin [50] with strong performance despite observed differences in cell image among the datasets. This further reinforces the suitability and representativeness of the engineered features used in the Explanation Module, ensuring that the model remains both interpretable and effective. These 2 datasets have the same cell types as the proprietary dataset except *large granular lymphocyte*. See Table 4 for detailed model performance.

The inference time from *Prediction* to *Explanation* modules averages at 3.99s (SD: 0.15s) on the proprietary dataset. The inference speed is further repeated on PBC dataset at 3.91s (SD: 0.14s) and Raabin dataset at 4.08s (SD: 0.21s). This is a significant improvement in efficiency in comparison to minimum 15 minutes our domain experts required for manual blood film examination. In general, a manual blood film examination could take 30 minutes [4].

## Explanation evaluation

### SHAP analysis

**Fig 9:**
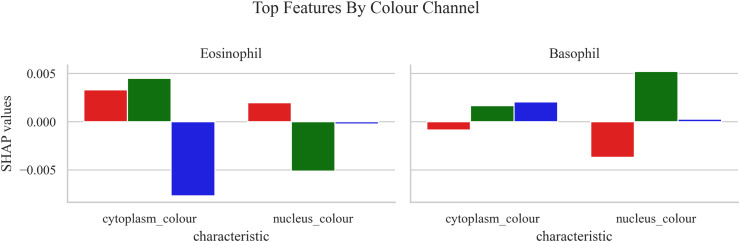
SHAP-based analysis of top colour features for eosinophils and basophils. Eosinophils, with their orange-reddish cytoplasm, show strong positive SHAP values for the red channel and negative values for the blue channel, indicating suppressed blue intensity. Basophils, which appear dark and bluish, exhibit predominantly positive SHAP values for blue and green channels, with negative contributions from the red channel, aligning with their staining characteristics.

Fig 9 illustrates the top colour features for eosinophils and basophils from SHAP. Eosinophils granules appear orange-reddish after staining due to their acidophilic structure. Basophil granules appear dark and bluish after staining. The figure shows that, base on the SHAP analysis, the red channel on the cytoplasm and nucleus exerts a positive influence on eosinophil. The large negative blue signal detected on the eosinophil cytoplasm indicates that blue has a strongly negative influence in predicting eosinophils. The SHAP analysis for basophils indicates that features related to the blue and green channels are predominantly positive, whereas those related to the red channel are negative. This shows that the blue and green channels are relatively more important than the red channel in predicting basophils.

**Fig 10:**
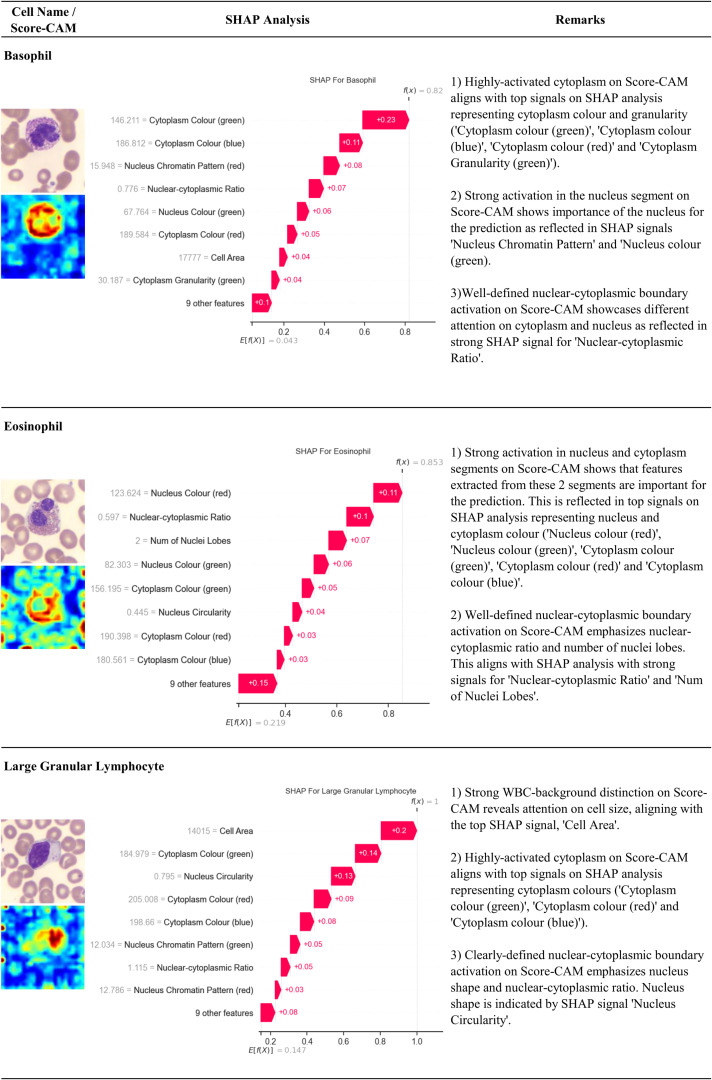
Alignment between YOLO classification and SHAP analysis for *basophil*, *eosinophil* and *large granular lymphocyte.*

**Fig 11:**
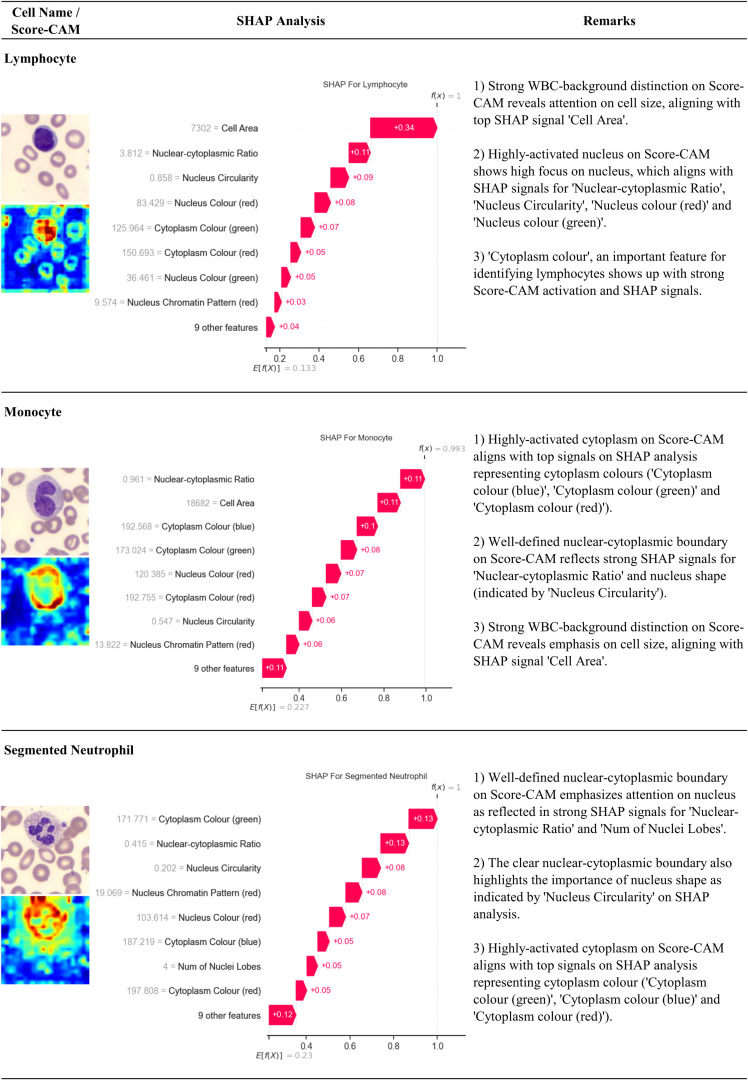
Alignment between YOLO classification and SHAP analysis for *lymphocyte*, *monocyte* and *segmented neutrophil.*

As a demonstration of alignment between YOLO and RF predictions, Figs 10 and 11 present a side-by-side comparison of Score-CAM heatmap against SHAP analysis. For better readability, the comparison for the 6 cells are split over 2 figures. Low activation on Score-CAM is indicated in blue in the heatmap. The colour transits from blue to red with increasing activation strength. With reference to *Basophil* in Fig 10 as an example, the basophil is clearly distinguished against the surrounding RBCs. The cytoplasm region is highly activated. The transition between nucleus and cytoplasm is also clearly demarcated, clearly demonstrating how each segment contributes differently to the prediction. SHAP analysis identifies the specific features responsible for the prediction and Score-CAM activation.

To a large extent, the strongly activated areas coincide with the top attributes identified by SHAP analysis. We further examined four scenarios defined by YOLO correctness with respect to ground truth and RF agreement with YOLO: (i) both YOLO and RF are correct, (ii) YOLO is correct but RF disagrees, (iii) YOLO is incorrect but RF agrees, and (iv) both models are incorrect. In the fully concordant scenario, the SHAP profile of the test sample is consistent with the global class-level SHAP profile (S4 Fig and S5 Fig in Supporting information section), with a mean RF residual of 0.0424 and an occurrence rate of 98.86%. In the remaining scenarios, which collectively account for 1.14% of cases, the mean RF residual is substantially higher. These findings indicate that large residuals are associated with weaker surrogate support for the YOLO decision and with a higher likelihood of disagreement or error. Based on the residual distribution of the full-agreement subset, we further derived a practical operating threshold of 0.1 using the Interquartile Range (IQR) method ($75^{th}percentile+1.5\times IQR$) (S6 Fig in Supporting information section). Predictions with residuals above this value may therefore warrant closer review.

### ROaR evaluation

To evaluate explanation performance, we conducted a feature ablation study using the RemOve And Retrain (ROaR) [56] technique on the proprietary dataset. ROaR is primarily a method to study the robustness of a machine learning model or to identify critical parts of the model design that influence the model prediction. The approach of this technique is to successively remove a component or feature from the model and then retrain the model to study the impact on accuracy at each removal.

In this study, ROaR is incorporated with a user study in which domain experts rank features by importance with respect to blood smear examination for each cell type. The haemotologists rank features based on visual interpretation. For example, cytoplasm colour is viewed as a whole and not by the three colour channels. Therefore, the removal of cytoplasm colour in the ROaR study refers to the removal of the feature for all 3 colour channels. We removed the highest user-ranked feature for each cell type and retrained the explanation model. This process was repeated iteratively, removing the next highest-ranked feature until the top five features were excluded. The evaluation considered up to half of the total features.

Table 6 presents the deterioration of precision and recall at each successive feature removal, as evaluated against the ground truth (human labels). The observed decline in model performance demonstrates that these engineered features, derived from segmentation, effectively capture clinically relevant characteristics. Furthermore, it confirms that the explanation model, built upon these features, successfully interprets YOLO’s predictions in a clinically meaningful way.

**Table 6:** ROaR evaluation: precision and recall at successive feature removal. Performance degrades mostly monotonically, indicating that the extracted features are meaningful and contribute consistently to the model’s predictions. The gradual decline suggests that these features effectively capture morphological characteristics.

Cell Type	Number of Features Removed					
	0	1	2	3	4	5
**Precision (Recall)**						
Basophil	1 (0.97)	0.99 (0.87)	0.97 (0.79)	0.91 (0.81)	0.78 (0.45)	0.79 (0.47)
Eosinophil	1 (0.99)	0.96 (0.98)	0.95 (0.95)	0.95 (0.95)	0.90 (0.92)	0.87 (0.87)
Large granular lymphocyte	0.98 (0.99)	0.95 (0.92)	0.93 (0.89)	0.92 (0.83)	0.86 (0.78)	0.72 (0.71)
Lymphocyte	1 (1)	1 (1)	1 (1)	1 (1)	1 (0.99)	0.98 (0.98)
Monocyte	0.99 (0.99)	0.99 (0.99)	0.98 (0.97)	0.88 (0.94)	0.86 (0.92)	0.78 (0.84)
Segmented neutrophil	0.99 (0.99)	0.99 (0.98)	0.98 (0.98)	0.94 (0.94)	0.92 (0.91)	0.92 (0.89)

It is observed that the classification performance for lymphocytes suffers the least degradation in comparison to the other WBCs in this study. This robustness can be attributed to the morphological extremities of lymphocytes, which are highly distinguishable. Specifically, lymphocytes possess the smallest size, the highest cell and nuclear circularity, and the highest nuclear-cytoplasmic ratio. Furthermore, their cytoplasm is characteristically agranular. These distinctive characteristics provide the model with a high degree of feature redundancy. Consequently, when the ROaR process ablates a top-ranked feature, the remaining morphological characteristics are sufficiently unique to maintain accurate identification.

### Model validation by clinicians

To further assess the clinical validity of the explanations, a model validation survey was conducted with 9 laboratory staff (haematologists and laboratory medical technologists - who all are trained and qualified to read and interpret peripheral blood films), using the Likert Scale [57]. A set of 18 images was selected, each displaying a model prediction alongside an explanation based on the interpretation of SHAP analysis. The haematologists and laboratory medical technologists were asked whether the identified features and accompanying explanation aligned with their own reasoning, with response options: *agree, somewhat agree, somewhat disagree*, and *disagree*. The average agreement rate among the respondents is 96.9% (SD: 8.35%), with individual agreement rates ranging from 94.4% to 100%. Of the 96.9% average agreement rate, 78.4% fully *agreed* with the explanations, while the remaining respondents selected *somewhat agree*. Agreement for each question falls between 8 and 9 out of 9 respondents. 1 question garners agreement for 6 out of 9 respondents.

Analysing the survey results further, we group the responses into 2 categories: *agree* and *somewhat agree* as positive; *disagree* and *somewhat disagree* as negative. Next, we compute the proportion of positive responses for each respondent. The mean of all the proportions is equivalent to the average agreement rate reported above as 96.9%. The 95% confidence interval calculated for this mean, based on the t-distribution with n−1=8 degrees of freedom, is [92.6%, 100%]. This provides strong evidence that agreement with the model prediction is substantially greater than a simple majority threshold of 50%.

For question-level agreement, we next determine the minimum number of respondents required to provide a positive response in order to be 95% confident that the majority agrees to the prediction of each survey question. Based on the proportion of respondents with positive responses, we compute the 95% confidence interval under the condition that the lower bound confidence limit must be above 50%. Using the Wilson confidence level [58], this is achieved if 8 out of 9 respondents agree with the prediction. 17 out of 18 survey questions meet this criterion, indicating 95% confidence that the majority of the respondents agreed with the model prediction. The single survey question that falls out of the criterion is due to respondents’ perception that the cell in question is a reactive lymphocyte, which in this study is labelled *large granular lymphocyte*.

In summary, in the context of SHAP interpretations based on engineered features trained on the model, clinicians generally agreed with the provided explanations. The results align with previous studies evaluating SHAP-based explanations through user studies involving clinicians [59]. These findings suggest that the model’s explanations are clinically viable and may serve as a valuable tool for decision support in haematological assessments.

## Conclusion

Interpretability is critical in AI-driven haematology, yet existing explanation techniques often fail to meet clinical needs. Feature-based methods such as Grad-CAM [14] and Score-CAM [33] generate heatmaps but do not provide morphological insights such as *size*, *shape*, and *granularity*, limiting their clinical applicability. Concept-based techniques like TCAV [41] identify image patterns but fail to capture attributes such as *circularity* and *area*, which are essential for blood cell classification. Recent works, such as WBCAtt [47], present datasets with detailed morphological annotations for a few predefined blood cell types, enabling supervised training of models using such annotations. However, their reliance on extensive manual labelling makes them labour-intensive and non-scalable to other cell types.

The proposed DREAM framework addresses these challenges by integrating deep learning with interpretable machine learning. Leveraging on established unsupervised machine learning model K-Means Clustering and modern unsupervised segmentation model SAM 2 [18], the fully automated segmentation module reduces the burden of manual annotation and alleviates the constraint of unavailable mask annotation data. From the segmented images, the framework seamlessly generates engineered features which closely mirror morphological properties. By coupling the YOLO classifier with a Random Forest explainer built on clinically engineered features derived from segmentation, the DREAM framework provides precise predictions which are verified by the RF explainer. This boosts confidence and trust in the predictions, while providing clinically meaningful explanations which align with the morphological criteria used by clinicians. The framework improves efficiency with significant reduction in time spent classifying each image.

While DREAM shows promising performance, several aspects of the current study define its scope and suggest directions for future work. At present, the framework focuses on a subset of common WBC subtypes and a limited number of datasets. Future work will potentially use advanced generative augmentation such as diffusion models or GANs to mitigate class imbalance. We will also extend the evaluation to rarer and morphologically atypical cells, such as myeloblasts and lymphoblasts, including cases that require recognition of subtle diagnostic cues such as chromatin structure, nucleoli, and lineage-specific inclusions such as Auer rods. Additionally, while the current engineered features capture major morphological properties, they do not yet fully represent these finer-grained attributes. We therefore plan to investigate richer feature representations, together with improved segmentation robustness in low-contrast cytoplasmic regions and pauci-stained boundaries.

The explainer relies on features derived from the KMeans–SAM2 segmentation pipeline, so we plan to further improve segmentation robustness, particularly for cells with pale cytoplasm, and to analyse how segmentation quality influences the resulting feature attributions. In addition, the RF component is used as a feature-level surrogate rather than a mechanistic model of YOLO’s internal representations, and future work will explore tighter integration with YOLO and alternative explainers. Finally, our clinician survey involved a modest number of experts, and expanding this evaluation across more institutions is an important next step. Together, these extensions are expected to further strengthen the robustness and clinical relevance of the proposed framework.

A limitation of this approach is that high agreement between the RF surrogate and YOLO reflects model faithfulness rather than guaranteed clinical correctness. Although our cross-dataset evaluation, spatial alignment, and residual-based error analysis provide supportive evidence, they do not fully exclude the possibility that the model may rely partly on dataset-specific shortcuts or clinically suboptimal cues.

To our knowledge, this is the first work to explain blood cell classification using clinically meaningful morphological concepts in an unsupervised manner, without human labelling. This study advances explainable AI in haematology, bridging the gap between model interpretability and clinical relevance. By achieving high classification performance while ensuring transparent decision-making, DREAM enhances trust in AI-assisted diagnostics, offering a scalable and effective solution for blood cell classification.

## Supporting information

S1 Fig Values of engineered features for proprietary and public datasets 1. Features: Cell Area, Cell Circularity, Nucleus Circularity, Nuclear-cytoplasmic Ratio and Number of Nuclei Lobes.
(EPS)

S2 Fig Values of engineered features for proprietary and public datasets 2. Features: Nucleus Colour (red, green, blue channels) and Nucleus Chromatin Pattern (red, green, blue channels).
(EPS)

S3 Fig Values of engineered features for proprietary and public datasets 3. Features: Cytoplasm Colour (red, green, blue channels) and Cytoplasm Granularity (red, green, blue channels).
(EPS)

S4 Fig SHAP analysis of test samples with incorrect and correct YOLO predictions. Four scenarios are presented: (i) both YOLO and RF are correct, (ii) YOLO is correct but RF disagrees, (iii) YOLO is incorrect but RF agrees, and (iv) both models are incorrect. In the optimal scenario with full agreement, top features on the SHAP analysis of the sample align with the global SHAP profile (S5 Fig), whereas, in the other scenarios, the features overlap is weaker and the existence of a strong signal from unrelated feature tips the prediction over, leading to wrong predictions.
(EPS)

S5 Fig Global class-level SHAP profile. (EPS)

S6 Fig Distribution plot and box plot of RF residuals. The distribution of full agreement subset (YOLO = GT, RF = YOLO) is significantly right-skewed. To account for this non-Gaussian behaviour, a statistical acceptance threshold was computed using the Interquartile Range (IQR) method (75*th percentile* + 1.5 *x IQR*), arriving at 0.1. With this acceptance threshold, the framework establishes a conservative safety margin that effectively isolates the high-confidence full agreement prediction cluster from all scenarios of model disagreement.
(EPS)

S1 Table Comparison of Random Forest (RF) residuals based on YOLO predictions. In the ideal scenario where YOLO predicted correctly and RF prediction aligns with YOLO prediction, mean RF residual is significantly lower than the suboptimal scenarios. In the evaluation, the ideal scenario has a good occurrence rate of 98.85%.
(EPS)
