# Supplementary material for: Aligning deep learning and interpretable models for blood cell classification: A dual-model framework for explainability
Source: PLOS Digit Health. 2026 Jul 30;5(7):e0001514. doi: 10.1371/journal.pdig.0001514 (PMC13422869; doi:10.1371/journal.pdig.0001514)
Supplement: Attachment — Submitted filename: Response to Reviewers.docx [file pdig.0001514.s009.docx]

# Reviewer Comments

## Response to the Academic Editor

**The work addresses an important clinical need for interpretable AI in hematology. While the technical execution demonstrates competence and the classification performance is strong, the central claim that the RF model 'explains' YOLO predictions requires significant revision.
All three reviewers have provided valid and constructive feedback. Reviewer 1's fundamental methodological concern is particularly important. I recommend Major Revision with the understanding that the authors should either (1) provide rigorous quantitative evidence that RF truly explains YOLO's reasoning, or (2) reframe their claims more modestly while still highlighting the framework's genuine value as a dual-classification system with interpretable outputs.**

**Response:** Thank you for your careful summary and for recognizing the clinical importance of interpretable AI in haematology. We fully agree with the central concern: our original wording could be interpreted as claiming that the Random Forest (RF) explains YOLO’s internal decision mechanism, which is stronger than what our current evidence can rigorously support.

Accordingly, in the revision we will adopt the second path you outlined and reframe our claims more modestly and precisely. Specifically, we will revise the abstract, introduction, and conclusion to state that the RF component functions as a concept-level surrogate trained to approximate YOLO outputs using clinically defined morphological features. The RF+SHAP results therefore provide interpretable, clinically grounded evidence that is consistent with (and predictive of) YOLO predictions, rather than a guaranteed mechanistic account of YOLO’s internal representations. To ensure this reframing is supported substantively, we will also strengthen the manuscript’s evaluation of surrogate faithfulness. Concretely, we will add representative failure-case analysis where $\epsilon$ is large, clarifying when explanations should be treated as lower-confidence.

With these changes, we will present DREAM explicitly as a dual-model system: (a) YOLO provides high-performance detection/classification, and (b) the RF-based surrogate provides transparent, clinically interpretable feature attributions aligned with YOLO’s outputs. We believe this addresses the reviewers’ methodological concern while accurately highlighting the framework’s practical value: pairing strong predictive performance with clinically meaningful interpretability.

## Reviewer 1

**This work proposes DREAM, an interpretable blood cell classification framework for blood smear examination, which uses YOLO to detect and classify cells, segment cells via K-means clustering and SAM 2, extracts clinically meaningful cell features, and then uses a Random Forest (RF) classifier to explain YOLO’s predictions. The YOLO model achieves good classification performance, while the RF explainer demonstrates good performance in replicating YOLO’s predictions. The authors claim that the results of the SHAP analysis of the explainer align with those of Score-CAM on YOLO, supporting the capacity of the RF explainer to provide interpretable explanations for YOLO.**

**The authors provide a good overview of past work on using explainable AI for blood cell classification. Standard explainability techniques, namely Score-CAM and SHAP, are employed for model explanations. The authors extended the SHAP formulation to include the discrepancy between the predictions of YOLO and the RF classifier, enabling the calculation of the RF model’s explanation for the YOLO model’s prediction. The adoption of unsupervised K-means clustering and SAM 2 for cell segmentation is appropriate and reduces the need for manual annotation, though more quantitative evaluation of the segmentation performance would be beneficial. The DREAM framework demonstrates excellent generalisability in the PBC and Raabin datasets. Model validation by clinicians was conducted, which provides a valuable quantitative assessment of the quality of the explanations generated by SHAP analysis.**

**However, the reviewer does not fully support the authors’ conclusion and assumptions. Despite achieving comparable accuracy to the YOLO classifier, the RF classifier utilises features that are not directly generated by YOLO. The morphological features are generated using segmentations obtained with K-means clustering and SAM 2, so they may no longer fully correlate with the features used by YOLO for prediction. Though the authors claim that the RF model’s SHAP analysis highlights regions and features that are also looked at by YOLO (shown by Score-CAM), there is no guarantee that the YOLO model and the RF model have similar decision-making mechanisms. Therefore, the reviewer does not think the statement that the YOLO model’s predictions are interpretable via the RF model is appropriate. Since the RF model also achieves good blood cell classification accuracy, the authors should further clarify their choice of using YOLO’s prediction instead of the RF model’s prediction for cell classification.**

**Please see below for detailed comments from the reviewer. Additionally, the authors could further polish the manuscript by adopting a more academic style of English.**

**Response:** We thank the reviewer for the thorough summary and constructive assessment of DREAM, including the clinical validation component. We agree with the reviewer’s key methodological concern: because the RF model is trained on externally extracted morphological features (from K-means + SAM2 segmentation) rather than YOLO’s internal representations, high agreement between RF and YOLO does not by itself guarantee identical decision mechanisms. At the same time, DREAM is explicitly designed to maximize faithfulness at the prediction level and to provide converging evidence that the clinical-feature explanations are meaningfully tied to YOLO’s own evidence. In particular, as detailed in our responses to Comments 12–14, we quantify RF–YOLO agreement and discrepancies, compare SHAP-based feature attributions with Score-CAM maps on YOLO, and incorporate clinician ratings of explanation quality, all supporting the view that the RF explanations are strongly aligned with YOLO’s predictive behavior, even if they are not a perfect mechanistic reconstruction.

In the revision, we will therefore reframe our claims to state that the RF model provides a concept-level surrogate that is predictive of and aligned with YOLO outputs, offering clinically meaningful evidence consistent with YOLO predictions rather than a mechanistic explanation of YOLO. We will add an explicit limitations paragraph clarifying this scope and the implications of using externally derived morphological features instead of YOLO’s internal feature space.

Regarding why we use YOLO predictions for classification rather than RF predictions, we will clarify the design rationale and the complementary roles of the two components. YOLO operates directly on the raw smear image to jointly perform detection and classification in an end-to-end manner, which is essential for practical deployment where cells must first be localized and where performance must remain robust under variations in staining, illumination, and morphology. In contrast, the RF model relies on segmentation-dependent morphological features, which can be sensitive to segmentation quality and may not fully capture the visual cues leveraged by a deep detector. We therefore present DREAM as a dual-component framework: YOLO provides the primary high-performance detection output, while the RF surrogate provides clinician-aligned, concept-level attributions that are predictive of YOLO outputs and serve as an interpretable rationale and complementary check. We revise the manuscript to articulate this trade-off explicitly and include additional discussion to make the roles and limitations of each component clear.

Major comments
Introduction
**1) Page 2, lines 9-10: It would be helpful if the authors could elaborate on the current challenges and limitations of manual blood smear examination, preferably with quantitative information. References should be provided.**

**Response:** Thank you for this helpful suggestion. We have expanded the description of current challenges and limitations of manual blood smear examination and now include quantitative information and supporting references. Specifically, we (a) report the proportion of samples that still require manual smear review despite automated haematologic analysers, and (b) provide data on the time burden and inter-observer variability of manual examination.

The edited paragraph is as follows:

“In routine hospital practice, initial screening is typically performed using automated haematologic analysers; however, these systems cannot fully replace manual blood smear examination. A previous study reported that 26.7% of specimens still required verification by manual smear review [3]. Manual blood smear examination involves visual inspection of red blood cells (RBCs), white blood cells (WBCs), and platelets under a microscope and classification according to their morphology (e.g., size, shape, colour, and granularity). This process is time-consuming, requiring on average about 30 minutes per specimen [4], and is subject to human bias and skill differences, resulting in documented inter-observer variability in examination results [5].”

[3] Novis, David A., Walsh, Molly, Wilkinson, David, St. Louis, Mary, and Ben-Ezra, Jonathon. Laboratory productivity and the rate of manual peripheral blood smear review: a College of American Pathologists Q-Probes study of 95 141 complete blood count determinations performed in 263 institutions. Archives of pathology & laboratory medicine; 2006.

[4] Pratumvinit, Busadee, Wongkrajang, Preechaya, Reesukumal, Kanit, Klinbua, Cherdsak, and Niamjoy, Patama. Validation and optimization of criteria for manual smear review following automated blood cell analysis in a large university hospital. Archives of Pathology & Laboratory Medicine; 2013.

[5] Fuentes-Arderiu, Xavier, Garc´ıa-Panyella, Mariona, and Dot-Bach, Dolors. Between-examiner reproducibility in manual differential leukocyte counting. Accreditation and quality assurance; 2007.

**2) Page 2, lines 38-39: Please give examples or provide further explanations. References should be provided.**

**Response:** Thank you for this helpful comment. We have expanded the discussion to provide concrete examples of how lack of transparency limits the adoption of deep learning in clinical practice, and how XAI can mitigate these concerns.

We have included references and updated the paragraph:

“The lack of transparency in deep learning models has led to substantial hesitation in adopting them for medical applications. To address this impediment, Explainable Artificial Intelligence (XAI) techniques have been explored to provide interpretable justifications for model predictions [11]. For example, in a survey with clinicians, respondents reported that trust in an AI system depends not only on its accuracy, but also on whether its explanations accord with clinical intuition during time-critical decision-making [12]. Furthermore, studies in high-risk medical environments have shown that integrating XAI methods—such as visual saliency maps or concise textual rationales—can increase user trust, reduce perceived risk, and improve willingness to rely on AI-generated decisions [13]. Such transparency is essential for the medical community to fully embrace AI for diagnostic applications.“

[12] Tonekaboni, Sana, Joshi, Shalmali, McCradden, Melissa D., and Goldenberg, Anna. What clinicians want: contextualizing explainable machine learning for clinical end use. Machine learning for healthcare conference. PMLR; 2019.

[13] Stegelmann, Mark, and Schlegel, Thomas. Explainable Artificial Intelligence in Health Care: How XAI Improves User Trust in High-Risk Decisions. In: International Conference on Human-Computer Interaction. Springer; 2022. p. 89–99.

Related work
**3) Page 4, lines 100-102: Please explain how the features were trained. This sentence is confusing.**

**Response:** Thank you for pointing this out. We have edited the sentences for clarity:

“Su et al. [22] first segmented the cellular components of WBCs and extracted geometric, colour, and textural features from these regions. These hand-crafted features were then used as inputs to train three classifiers: a support vector machine (SVM), a hyperrectangular composite neural network (HRCNN), and a multilayer perceptron (MLP).”

**4) Page 4, lines 107-109: Please include references and describe past research that adopted ResNet and CNN for blood cell classification.**

**Response:** Thank you for this suggestion. We have expanded the related work section to more clearly describe prior studies that adopted convolutional neural networks for blood cell classification:

“In deep learning approaches, both feature extraction and classification are learned jointly within the network. Commonly used convolutional architectures include VGG and ResNet variants. For example, Asghar et al. [25] employed transfer learning with pre-trained CNNs for classifying basophils, eosinophils, lymphocytes, monocytes and neutrophils, reporting average accuracies of 96.86% for VGG16, 92.40% for VGG19, 95.56% for ResNet50, 91.60% for ResNet101, and 96.00% for ResNet152. In their setting, the models were trained and evaluated on full-field images containing both WBCs and background. Such configurations can be sensitive to variations in image composition; for instance, performance may degrade when a test image contains multiple WBCs or substantially different red blood cell (RBC) density as compared to the training images, potentially challenging generalisation in real-world smear examinations.”

[25] Asghar, Rabia, Kumar, Sanjay, and Hynds, Paul. Automatic classification of 10 blood cell subtypes using transfer learning via pre-trained convolutional neural networks. Informatics in Medicine Unlocked; 2024.

**5) Page 4, lines 119-121: Since the authors state that explaining DL model predictions “proved” to be difficult, please provide some proof and references.**

**Response:** Thank you for the comment. We have revised the text to ground the claim in the established XAI literature, with explicit references:

“While classical machine learning models (linear models, decision trees) often permit relatively direct inspection of their parameters or decision rules, explaining the predictions of deep learning models remains challenging due to their high-dimensional, deeply layered, and non-linear architectures, in which intermediate representations do not correspond to human-interpretable concepts in a straightforward manner [27]. In the medical domain, this ‘black-box’ nature has shown to hinder traceability and the rigorous validation required for clinical safety and regulatory acceptance, contributing to clinicians’ reluctance to rely on such systems in practice [11].”

[27] Arrieta, Alejandro Barredo, et al. Explainable Artificial Intelligence (XAI): Concepts, taxonomies, opportunities and challenges toward responsible AI. Information fusion; 2020.

**6) Table 1: In the “Method” column, it would be helpful to provide more details on the classical ML methods adopted.**

**There are many other studies on blood cell classifications using classical ML or DL methodologies. Here are some examples:**

**• Lamberti 2021** [**https://doi.org/10.1016/j.cmpbup.2021.100023**](https://doi.org/10.1016/j.cmpbup.2021.100023)

**• Zou et al. 2024 [https://doi.org/10.1016/j.bspc.2024.106820](https://doi.org/10.1016/j.bspc.2024.106820)**

**• Sherpa et al. 2025 [https://doi.org/10.1002/ima.70084](https://doi.org/10.1002/ima.70084)**

**The authors also mentioned other studies on blood cell classification in the “Explainable AI” subsection. Could the authors explain why they include these specific studies in the table? It would be more informative to include the results of more studies that proposed explainable/interpretable DL methods.**

**Response:** Thank you for the comment and for sharing additional information. We have revised Table 1 so that the “Method” column now lists the specific classical algorithms used (e.g., Naïve Bayes, Decision Tree, k-Nearest Neighbour) instead of the previous generic label “classical ML,” and we have updated the remarks to briefly describe the corresponding feature sets. We also expanded the related work section to cite additional recent ML/DL methods for WBC classification, including 2 of the works suggested by the reviewer (authors Zou and Sherpa). Regarding the work by Lamberti, while it classifies RBCs, WBCs, and platelets, it does not differentiate WBC subtypes. We have therefore referenced it in the introduction in the context of broader blood cell analysis."

Table 1 is intended as a representative summary for quantitative performance comparison across different modelling strategies. Most studies that propose explainable DL methods rely on heatmap-based XAI to highlight image regions that influence the prediction. These visual maps are generally qualitative; they do not explicitly specify which morphological properties are responsible for the highlighted regions. For example, if the nucleus area is emphasized on a heatmap, it remains unclear whether the model is responding to its size, contour irregularity, or lobulation. As these saliency maps cannot be directly quantified at the level of explicit morphological features, they cannot be compared numerically with our framework, which identifies and scores specific morphological properties. We therefore discuss these explainable DL methods in the text, while keeping Table 1 focused on comparable classification performance.

Table 1 before change:

Table 1 after change:

The study by Lamberti is added into Introduction:

“A similar study by Lamberti [8] used SVM with a polynomial kernel to classify RBCs, WBCs and platelets using shape-based features, such as eccentricity, extracted from segmented cells.”

**7) Page 5, lines 134-136: CAMs do not necessarily adopt a red to blue colour scheme.**

**Response:** Thank you for pointing this out. We agree that CAMs are not restricted to a specific red–to–blue colour scheme. We have revised the sentence to describe CAMs in general terms and present red–to–blue only as an example of a commonly used colour map. The updated text reads:

“CAMs are heatmaps that encode the influence of different image regions on the model’s prediction through colour intensity. For example, a commonly used colour scheme maps regions of highest influence to bright red, with intensity gradually decreasing through yellow and green and finally to deep blue for regions of minimal influence.”

Methodology

**8) Prediction module: It would be helpful to explain why YOLO is selected for prediction.**

**Response:** Thank you for the suggestion. We have expanded the description of the prediction module to explain our choice of YOLO in terms of both architecture and practical deployment considerations. In particular, we now emphasise its single-stage design, speed, ability to handle multiple cells per image, robustness to background artefacts, and prior successful use in medical imaging:

“The prediction module is implemented with YOLO11s, a single-stage detector that directly predicts bounding boxes and class probabilities from the full image in a single forward pass [16]. Compared with two-stage detectors, this design offers substantially faster inference, which is important for time-critical, high-throughput clinical laboratory workflows [52]. YOLO’s multi-scale feature maps also capture contextual information around each cell, improving robustness against misclassifying background artefacts as WBCs and allowing multiple WBCs in the same field of view to be detected and classified simultaneously. The suitability of YOLO for medical imaging has been demonstrated in several applications, including real-time WBC detection and malaria parasite detection [53,54], supporting its use as the backbone prediction network in DREAM.

We compared YOLO8 and YOLO11 across different batch sizes (32, 64, and 128) and different model sizes (``s'' & ``m''). Performance is similar for different models, model variants and batch sizes. YOLO11s (batch size 128) is selected for its efficiency. The bounding box result from the prediction is used to crop out the detected WBC in preparation for segmentation. This helps to reduce background noises from interfering with segmentation performance. “

[52] Alam, Mohammad Mahmudul, Islam, Mohammad Tariqul. Machine learning approach of automatic identification and counting of blood cells. Healthcare Technology Letters. 2019;6(4):103–108.

[53] Eamkong, Anoma, Pintavirooj, Chuchart, Treebupachatsakul, Treesukon. Real-Time White Blood Cell Classification with YOLO. 2025 17th Biomedical Engineering International Conference (BMEiCON). 2025:1-5.

[54] Lufyaila, Beston, Mgawe, Bonny, Sam, Anael. Fine-tuned YOLO-based deep learning model for detecting malaria parasites and leukocytes in thick smear images: A Tanzanian case study. Machine Learning with Applications. 2025;100687.

**9) Segmentation module: Some details on prompt optimisation (lines 212-213) are preferred. Also, please provide more descriptions of the fixed positions and the random positions. A figure or table would be helpful.**

**Response:** Thank you for this helpful comment. We have expanded the description of the prompt optimisation strategy in the Segmentation module and added a new figure (Fig. 3) to illustrate the fixed and random prompt locations:

“For SAM2-based segmentation, we use 13 point prompts per detected cell. Nine fixed prompts are placed within the YOLO bounding box so that, given the centered cell, they reliably fall inside the cell and provide consistent coverage. Four additional random prompts are sampled from pixels in the cytoplasm cluster identified by K-means to refine cytoplasmic boundaries. Figure 3 illustrates these fixed (red ‘X’) and random (blue ‘X’) prompt positions for an eosinophil as an example.”

Results

**10) Classification model: Have the authors attempted other augmentation methods, such as small scaling, translation and shearing?**

**Response:** Thank you for this suggestion. In our current study, the augmentation pipeline includes horizontal/vertical flips, small rotations, and colour-balance variations, but we did not incorporate scaling, translation, or shearing. We agree that mild geometric transformations could further improve robustness to variability in cell size and position. For example, helping the model better distinguish blasts from mature lymphocytes that have similar shapes by encouraging reliance on finer nuclear and textural cues. However, as our present classifier already achieves very high precision and recall across classes (Table 4), it is difficult to quantify the incremental benefit of additional augmentation on this dataset. We will include augmentation techniques such as small scaling, translation and shearing in our future work to expand this study to include more WBC subtypes.

Classification results (Table 4):

**11) Segmentation and feature generation: Would it be possible to quantitatively assess the performance of the segmentation method by manually creating some ground truth segmentations and comparing the predictions with the ground truths?**

**Response:** Thank you for the helpful suggestion. We quantitatively evaluated our segmentation method on a public WBC dataset that provides expert-annotated nucleus and cytoplasm ground-truth masks. We have added these results and example images to the revised manuscript.

Under ‘Materials and methods’ Section / ‘Datasets’ Sub-section:

“The segmentation module is evaluated on Mendeley public dataset [51]. The dataset consists of 400 120-by-120 pixels microscopic images of unlabelled WBC with corresponding nucleus and cytoplasm masks. The segment masks serve as ground truths to measure against the performance of our segmentation module.”

Under ‘Results’ Section / ‘Segmentation and feature generation’ Sub-section:

“The segmentation module was quantitatively evaluated on the Mendeley public dataset [51], which includes expert-annotated nucleus and cytoplasm masks. Our KMeans–SAM2 pipeline was applied to each cell, and the predicted nucleus and cytoplasm segments were compared against the corresponding ground truths using IoU and Dice coefficients. Table 5 shows the IoU and DICE results. Intersection of segments produced by our segmentation module and the ground truths achieves IoU above 85% and DICE above 91%. Representative examples are shown in Fig. 5. While the overall agreement is high, cytoplasm segmentation is slightly less accurate, particularly for cells with pale cytoplasm that are harder to distinguish from the background. To ensure reliable feature extraction, we retain only cells whose segmentation quality (IoU/Dice) lies within one standard deviation of the mean when training the explanation model.”

[51] Zheng, Xin. Data for: Fast and robust segmentation of cell images by self-supervised learning. Mendeley Data. 2018;V1. doi: 10.17632/w7cvnmn4c5.1.

Results: Table 5

Segmentation images: Figure 5

Explanation evaluation

**12) It is nice to see that the RF classifier’s explanations align with YOLO’s Score-CAM heatmaps. However, since the evaluation is qualitative and the morphological features are not directly generated by YOLO, the reviewer doubts whether the authors can confirm that the explainer successfully interprets YOLO’s prediction.**

ROaR evaluation

**13) Please elaborate on why this evaluation confirms that the explainer successfully interprets YOLO’s predictions.**

Conclusion

**14) Again, could the authors really confirm that the RF classifier explains the decision-making of the YOLO classifier?**

**Response to points 12, 13 and 14:**

We appreciate the reviewer’s concern. We acknowledge an important limitation: because the explainer operates on engineered morphological features derived from an external segmentation pipeline (K-means + SAM2), these features are not direct internal representations used by YOLO. Therefore, we cannot claim a formal guarantee that the RF surrogate reproduces YOLO’s full decision-making mechanism in every case. However, our framework is designed to maximize faithfulness at the prediction level and to provide converging evidence that the clinical-feature explanations are meaningfully tied to YOLO’s own evidence:

First of all, our explainer is explicitly tied to the base model: it is trained to predict/approximate YOLO’s outputs using clinically-defined morphological features as inputs. This design aims to explain YOLO’s decisions at the concept/clinical feature level rather than producing an independent diagnosis. The bounding box result derived from YOLO prediction is used to crop out the detected WBC. The cropped image is then used to perform segmentation from which engineered features are extracted. This further strengthens the relationship between the RF model and the YOLO model.

Secondly, CAM-based localization is widely used as a sanity check to verify that the network is focusing on biologically significant regions rather than learning spurious correlations from the image background [14]. For instance, if a CAM heatmap shows strong influence from the background region, the prediction is likely unreliable. Conversely, if a CAM heatmap shows strong influence over the cell region, the prediction is more likely to be reliable However, the CAM heatmaps only show regions of high influence to the prediction and do not identify the specific morphological properties responsible. To check that these feature-level explanations relate to YOLO’s own evidence, we extract YOLO activation regions using Score-CAM and compare them with the key medical features identified by the explainer. Therefore, we use it as complementary evidence: the strong correlation we observe supports that the explainer reflects YOLO’s reasoning.

Thirdly, we compare the performance of RF trained on ground truth label and YOLO prediction. Please refer to Table 4 below for results in groups RF-GT and RF-Y11. While both groups show good performance, the 2 results are different, showing that RF is connecting directly with YOLO.

Fourthly, since these top features identified by the RF explainer are responsible for the prediction, removing these features progressively weakens the RF explainer’s ability to correctly classify the cell, accounting for performance deterioration. This shows that the engineered features are highly representative.

Finally, we also validate the clinical plausibility of the explanations through expert review in our survey, which supports that the highlighted morphological properties align with domain reasoning, even though this does not by itself prove internal-mechanism equivalence.

To address this limitation more fundamentally, we will focus on strengthening the methodological coupling between the explanation pipeline and YOLO in future work.

[14] Selvaraju, Ramprasaath R, Cogswell, Michael, Das, Abhishek, Vedantam, Ramakrishna, Parikh, Devi, Batra, Dhruv. Grad-cam: Visual explanations from deep networks via gradient-based localization. Proceedings of the IEEE international conference on computer vision. 2017:618–626.

Classification results (Table 4):

**15) Some discussion on the study’s limitations would be helpful.**

**Response:** Thank you for this suggestion. We have added a short paragraph in the Discussion that briefly outlines the current scope of our study and the main directions for future work, including broader WBC coverage, improved segmentation, tighter coupling between YOLO and the explainer, and larger-scale clinical validation:

“While DREAM shows promising performance, several aspects of the current study define its scope and suggest directions for future work. At present, the framework focuses on a subset of common WBC subtypes and a limited number of datasets. Future work will extend the evaluation to rarer and morphologically atypical cells, potentially using advanced generative augmentation such as diffusion models or GANs to mitigate class imbalance. The explainer relies on features derived from the KMeans–SAM2 segmentation pipeline, so we plan to further improve segmentation robustness, particularly for cells with pale cytoplasm, and to analyse how segmentation quality influences the resulting feature attributions. In addition, the RF component is used as a feature-level surrogate rather than a mechanistic model of YOLO’s internal representations, and future work will explore tighter integration with YOLO and alternative explainers. Finally, our clinician survey involved a modest number of experts, and expanding this evaluation across more institutions is an important next step. Together, these extensions are expected to further strengthen the robustness and clinical relevance of the proposed framework.”

Minor comments

Introduction

**16) Page 2, lines 14-15: The nucleus is a cell organelle, so the authors should remove the “cell” before “boundaries”. Also, please change “classify” to the past tense.**

**Response:** Thank you for the comment. The lines have been edited accordingly:

“In one such early work, Street et al. [6] demarcated boundaries of cell nuclei and extracted features such as perimeter, area, compactness and classified the cells based on these extracted features using Multi-surface Method to define separating planes.”

**17) Page 2, lines 22-29: Some references for the statements would be useful.**

**Response:** Thank you for pointing this out. We have added a reference to support the statements about deep learning models and their capacity to model complex, non-linear image patterns:

“Deep learning models, on the other hand, are designed to process images at the pixel level, capturing both spatial and hierarchical relationships. They are also capable of modelling complex data structures and learning non-linear patterns that traditional methods may struggle to represent [9].”

[9] LeCun, Yann, Bengio, Yoshua, and Hinton, Geoffrey. Deep learning. Nature; 2015.

Related work

**18) Page 3, lines 93-94: Could the authors provide examples of the features that were extracted on the nucleus? Would “from the nucleus” or “of the nucleus” be more appropriate?**

**Response:** Thank you for the helpful comment. We have clarified both the type of features and the phrasing:

“Gautam et al. [20] extracted geometric features from the nucleus, including area, perimeter, circularity, and eccentricity, and achieved 80.88% accuracy with a Naive Bayes classifier.”

**19) Page 4, line 104: Please include the unabbreviated form of ANN.**

**Response:** Thank you for the comment. Unabbreviated form of ANN has been included:

“Manik et al. [23] and Hegde et al. [24] subsequently used artificial neural networks (ANNs) to classify cells based on extracted features, achieving accuracies of 98.90% and 99.00% respectively.”

**20) Page 4, line 127: “Leveraged on ResNet” does not sound grammatically correct.**

**Response:** Thank you for highlighting this. We have revised the sentence to improve clarity and grammar:

“Similarly, Chen et al. [38] incorporated Grad-CAM into their proposed CNN architecture, which was built on ResNet and DenseNet backbones and integrated with spatial and channel attention modules, while Katar et al. [34] used Score-CAM to explain predictions from a Vision Transformer model.”

**21) Page 5, lines 136-137: Please provide references for the techniques.**

**Response:** Thank you for the suggestion. References have been included:

“There are many other techniques that engage CAMs, such as Layer-CAM [39] and Ablation-CAM [40]. However, all these techniques are not able to infer the morphological properties responsible for the predictions.”

[39] Jiang, P. T., Zhang, C. B., Hou, Q., Cheng, M. M., and Wei, Y. LayerCAM: Exploring Hierarchical Class Activation Maps for Localization. IEEE Transactions on Image Processing. 2021;30:5875–5888.

[40] Ramaswamy, H. G., et al. Ablation-cam: Visual explanations for deep convolutional network via class-specific ablation. IEEE Winter Conference on Applications of Computer Vision (WACV). 2020:3031–3040.

**22) Page 5, line 158: There seems to be a grammar mistake.**

**Response:** Thank you for the comment. The lines have been edited for clarity:

“Since a combination of these morphological properties is essential for differentiating the WBCs, the lack of discrete numerical values for comparison limits the overall explainability power and makes it challenging to scale to additional cell types.”

Methodology

**23) Feature generation module: More details on how the features were derived and calculated computationally would be useful.**

**Response:** Thank you for the helpful suggestion. Further explanations on deriving the features have been included:

“The Feature Generation module computes quantitative descriptors that capture the clinical characteristics of each cell type. Following the feature design in [55], we defined 18 engineered features, as summarised in Table 3. With the exception of circularity, all features are computed directly from the nucleus and cytoplasm segments. For example, nuclear and cytoplasmic sizes are obtained by computing the pixel area of each segment using the contour-area function in the OpenCV library, and total cell area is defined as the sum of these two components. Chromatin pattern and cytoplasmic granularity are quantified by the standard deviation of colour intensity within the nucleus and cytoplasm segments respectively; this statistic serves as a proxy for textural heterogeneity, distinguishing, for instance, the highly granular cytoplasm of basophils from the more uniform cytoplasm of monocytes. Circularity is derived from the area and perimeter of each segment using Equation (1). In this work, the terms ‘cell shape’ and ‘nucleus shape’ refer to circularity, where a value of 1 corresponds to a perfect circle.”

**24) Table 4: It would be useful to have one or two more decimal places for the performance metrics.**

**Response:** Thank you for the suggestion. We have increased the precision of the performance metrics in Table 4 from two to four decimal places to provide more detailed reporting.

Table 4 Before change:

Table 4 After change:

**25) Page 10, lines 300-302: Please provide a reference for the time required for manual blood film examination.**

**Response:** Thank you for this suggestion. The lines have been edited:

“This is a significant improvement in efficiency in comparison to minimum 15 minutes our domain experts required for manual blood film examination. In general, a manual blood film examination could take 30 minutes [4].”

[4] Pratumvinit, Busadee, Wongkrajang, Preechaya, Reesukumal, Kanit, Klinbua, Cherdsak, and Niamjoy, Patama. Validation and optimization of criteria for manual smear review following automated blood cell analysis in a large university hospital. Archives of Pathology & Laboratory Medicine; 2013.

ROaR evaluation

**26) It is interesting to see that the classification accuracy of lymphocytes remains high after removing the top five features. Some hypotheses on potential reasons would be beneficial.**

**Response:** Thank you for the comment. An explanation on the model robustness for lymphocytes has been included:

“It is observed that the classification performance for lymphocytes suffers the least degradation in comparison to the other WBCs in this study. This robustness can be attributed to the morphological extremities of lymphocytes, which are highly distinguishable. Specifically, lymphocytes possess the smallest size, the highest cell and nuclear circularity, and the highest nuclear-cytoplasmic ratio. Furthermore, their cytoplasm is characteristically agranular. These distinctive characteristics provide the model with a high degree of feature redundancy. Consequently, when the ROaR process ablates a top-ranked feature, the remaining morphological characteristics are sufficiently unique to maintain accurate identification.”

## Reviewer 2

**This manuscript proposes DREAM, a dual-model framework combining YOLO for blood cell classification with a Random Forest surrogate trained on engineered morphological features to provide SHAP-based explanations. The approach aims to bridge deep learning performance with clinically meaningful interpretability. The evaluation includes multi-dataset testing, CAM–SHAP alignment, ROaR feature ablation, and a clinician user study.**

**Strengths:**

**1.Innovative integration of deep learning and a feature-based surrogate to achieve clinically interpretable outputs.**

**2.Strong YOLO performance across proprietary and public datasets.**

**3.Well-designed explainability pipeline (SHAP, Score-CAM, ROaR).**

**4.Meaningful engagement with clinical experts; explanations rated highly (96.9%).**

**5.Clear writing and solid methodological structure.**

**Major Weaknesses (Require Minor Revision):**

**1) Surrogate model explains YOLO, not the ground truth.**

**Since the Random Forest surrogate is trained on YOLO predictions, SHAP reflects YOLO’s internal decision logic rather than clinical ground truth. Please include a brief analysis of several YOLO misclassified cases, showing how SHAP behaves when YOLO is incorrect and discussing the meaning of the residual term ε (i.e., how often it becomes large and what that implies about explanation reliability).**

**Response:** Thank you for this insightful comment. We would like to clarify our aim: the goal of DREAM is to characterise the decision logic of the deep learning model (YOLO) in an interpretable clinical feature space, rather than to re-derive the underlying medical diagnostic rules, which are already well understood by haematologists. In this setting, the SHAP analysis is intended to reveal which morphological features drive YOLO’s predictions, as captured by the RF surrogate.

To better support this claim, we added an analysis that separates cases where YOLO is correct from cases where YOLO is incorrect, and examined how the RF explainer behaves in each group. S4 Fig in ‘Supporting information’ section presents the representative examples. We add an analysis of 1 group of YOLO failure cases and 1 group of success cases. Images 1 and 2 illustrate cases where YOLO predicts the wrong class with low confidence, and the RF prediction probabilities are correspondingly modest. Images 3 and 4 show cases where YOLO predicts right. We can observe that the prediction confidence of YOLO is always consistent with that of RF. The associated SHAP plots indicate that, when YOLO is confident and correct, the dominant features for the test image are consistent with the global class-level SHAP profile (S5 Fig), whereas wrong cases show weaker and less consistent support.

The global SHAP profile shows that the top 3 features are cell area, cytoplasm colour and nucleus circularity. These coincides with the top 3 features reported in the SHAP analysis in the instances where YOLO predicted correctly. Conversely, in the incorrect YOLO prediction instances, the top features in SHAP analysis do not conform to the global SHAP analysis.

S4 Fig. SHAP analysis of test samples with incorrect and correct YOLO prediction.

S5 Fig: SHAP analysis for large granular lymphocyte.

We also provide a quantitative summary in S1 Table in ‘Supporting information’ section. For samples where YOLO predicts correctly, the mean RF residual is 0.0493 and YOLO–RF agreement in predicted class is 99.02%. For samples where YOLO predicts incorrectly, the mean RF residual increases to 0.6222 and YOLO–RF agreement falls to 33.33%. Thus, large residuals are associated with lower RF support for YOLO’s decision and more frequent disagreement in predicted class. This suggests that the RF surrogate not only approximates YOLO’s behaviour when YOLO is reliable, but also tends to flag potential YOLO errors by producing a poor fit, which we now emphasize in the revised manuscript.

The explanation has been included:

“Additionally, we examined the SHAP analysis of correct and incorrect YOLO prediction against ground truth. Top features on the SHAP analysis of incorrectly predicted test samples are different from the expected top features based on the ground truth global class-level SHAP profile, whereas those from correctly predicted test samples conformed to the global class-level SHAP profile (S4 and S5 Figs in Supporting information section) with mean RF residual of 0.0493 and YOLO–RF agreement of 99.02%. For samples where YOLO predicts incorrectly, the mean RF residual increases to 0.6222 and YOLO–RF agreement falls to 33.33%. Thus, large residuals are associated with lower RF support for YOLO’s decision and more frequent disagreement in predicted class. This suggests that the RF surrogate not only approximates YOLO’s behaviour when YOLO is reliable, but also tends to flag potential YOLO errors by producing a poor fit as shown in S1 Table in Supporting information section.”

**2) No quantitative segmentation validation.**

**The segmentation module (K-means + SAM2) is essential for feature extraction but is evaluated only visually. Please provide minimal quantitative validation (e.g., IoU/Dice or inter-annotator agreement on a small subset), or explicitly acknowledge this as a limitation and discuss its possible impact on downstream features.**

**Response:** Thank you for this important comment. We have added quantitative validation of the K-means + SAM2 segmentation module on a public dataset with ground-truth masks.

Under ‘Materials and methods’ Section / ‘Datasets’ Sub-section:

“The segmentation module is evaluated on Mendeley public dataset [51]. The dataset consists of 400 120-by-120 pixels microscopic images of unlabelled WBC with corresponding nucleus and cytoplasm masks. The segment masks serve as ground truths to measure against the performance of our segmentation module.”

Under ‘Results’ Section / ‘Segmentation and feature generation’ Sub-section

“The segmentation module was quantitatively evaluated on the Mendeley public dataset [51], which includes expert-annotated nucleus and cytoplasm masks. Our KMeans–SAM2 pipeline was applied to each cell, and the predicted nucleus and cytoplasm segments were compared against the corresponding ground truths using IoU and Dice coefficients. Table 5 shows IoU and DICE results. Intersection of segments produced by our segmentation module and the ground truths achieves IoU above 85% and DICE above 91%. Representative examples are shown in Fig. 5. While the overall agreement is high, cytoplasm segmentation is slightly less accurate, particularly for cells with pale cytoplasm that are harder to distinguish from the background. To ensure reliable feature extraction, we retain only cells whose segmentation quality (IoU/Dice) lies within one standard deviation of the mean when training the explanation model.”

[51] Zheng, Xin. Data for: Fast and robust segmentation of cell images by self-supervised learning. Mendeley Data. 2018;V1. doi: 10.17632/w7cvnmn4c5.1.

Results: Table 5

Segmentation images: Figure 5

**3) Limited morphological feature set.**

**Discuss how more advanced descriptors (chromatin clumping, nucleoli, vacuolation, toxic granules, blast morphology) could be incorporated in future work.**

**4) Generalizability to pathological cells unclear.**

**Acknowledge the limitation of using primarily normal WBCs and outline plans for extension to abnormal/pathological samples.**

**5) User study limitations.**

**Small cohort (n=9) and limited cases; clarify limitations and propose improved future study designs.**

**Response to points 3, 4 and 5:** Thank you for these constructive suggestions. We have included more information for our future work which seeks to resolve current limitations:

“While DREAM shows promising performance, several aspects of the current study define its scope and suggest directions for future work. At present, the framework focuses on a subset of common WBC subtypes and a limited number of datasets. Future work will extend the evaluation to rarer and morphologically atypical cells, potentially using advanced generative augmentation such as diffusion models or GANs to mitigate class imbalance. The explainer relies on features derived from the KMeans–SAM2 segmentation pipeline, so we plan to further improve segmentation robustness, particularly for cells with pale cytoplasm, and to analyse how segmentation quality influences the resulting feature attributions. In addition, the RF component is used as a feature-level surrogate rather than a mechanistic model of YOLO’s internal representations, and future work will explore tighter integration with YOLO and alternative explainers. Finally, our clinician survey involved a modest number of experts, and expanding this evaluation across more institutions is an important next step. Together, these extensions are expected to further strengthen the robustness and clinical relevance of the proposed framework.”

Minor Weaknesses

**1) Provide key RF hyperparameters (e.g., number of trees, depth).**

**Response:** Thank you for this comment. We now report the main Random Forest hyperparameters used in the explanation module.

“The Explanation module consists of a Random Forest classifier (150 trees, maximum depth 15) trained on the engineered morphological features, together with SHAP analysis for explanation generation.”

**2) Including confusion matrices for YOLO and RF would improve transparency.**

**Response:** Thank you for the suggestion. To improve transparency of the classification behaviour, we have added confusion matrices for both YOLO and the RF surrogate.

“Fig 4 shows the confusion matrices for YOLO and for the RF classifier on the test set. These matrices allow inspection of per-class performance and error patterns, and illustrate that the RF surrogate closely follows YOLO’s predictions while providing an interpretable feature-based rationale.”

Fig 4. Confusion matrices of YOLO11 against ground truth and RF against YOLO11.

**3.CAM–SHAP figure captions could be slightly expanded for clarity.**

**Response:** Thank you for the suggestion. We have improved the appearance of the figures.

Fig 9. Alignment between YOLO classification and SHAP analysis for basophil, eosinophil and large granular lymphocyte

Fig 10. Alignment between YOLO classification and SHAP analysis for lymphocyte, monocyte and segmented neutrophil

Recommendation
Minor Revision
**The manuscript is strong and suitable for publication after addressing the points above.**

## Reviewer 3

**In this manuscript, Yu Cheong et al describe the DREAM framework they constructed that accurately classifies WBCs from peripheral blood smear images. DREAM consists of the prediction module (YOLO object detection and classification model), segmentation module that crops nuclei and cytoplasm form individual cells, feature generation module that extracts pre-specified engineered features such as RGB channels and geometric/morphologic cell features, and finally, explanation module, that uses RF model with SHAP explanations of individual features. The model was compared to ground truth established by manual labeling of the images by hematology experts.**

**There are numerous publications on blood cell classification models achieving very good accuracy, however, the main value of the dual model approach described in this manuscript is interpretability of the deep learning model. YOLO predictions and SHAP feature analysis of the explanation model predictions aligned at the same morphologic regions, which is encouraging. In addition, authors performed a feature ablation analysis (ROaR technique) with successive engineered feature removal, testing the model's accuracy with each removed feature. This showed expected performance decline with each removed feature, confirming that the model captures and depends on clinically relevant cell characteristics.**

**The manuscript is well-written and easy to follow.**

**All proposed figures and tables are relevant and consistent with the content of the manuscript.**

**Remarks:**

**1. My main concern are small validation sets (9,730 and 2,670 of cell images), although it is encouraging that accuracy was retained despite different staining methods and image sizes in the validation sets. Did authors perform any color corrections or other image alterations on the validations sets?**

**Response:** Thank you for this thoughtful comment. We agree that larger, multi-centre validation would further strengthen the evidence for generalisability. In our current study, for each dataset the YOLO and RF models are trained and validated on that dataset’s own training split, and then evaluated on a held-out test split (9,730 and 2,670 cells respectively). We did not apply any stain normalisation, colour correction, or other image alterations specifically to the validation or test sets.

**2. In terms of clinical applicability of this model and using it as an AI assistant tool for hemato-pathologists, images of peripheral blood smears used prospectively would need to be cropped first? What tool do the authors propose to prepare/crop images of individual cells for analysis? How much additional time would this take?**

**Response:** Thank you for raising this practical point. In our current clinical setting, peripheral blood smears at Tan Tock Seng Hospital are already digitised and pre-processed by a digital morphology analyser (CellaVision DI-60). This system automatically detects WBC candidates on the smear and exports cropped single-cell images; according to our collaborators, this scanning and cropping step typically takes less than one minute per slide. DREAM is designed to operate on these pre-cropped cell images, so no additional manual cropping is required. In practice, our framework would be implemented as a downstream software module within the existing digital workflow (for example, integrated with CellaVision DI-60, and potentially with the Blade system in future once more WBC subtypes are covered), adding only the model inference time, which is small relative to the slide scanning process. We have added a sentence in the section on data acquisition:

“Peripheral blood smears are digitised using a CellaVision DI-60 analyser, which automatically detects WBC candidates and exports cropped single-cell images, typically within one minute per slide. DREAM operates on these pre-cropped images as a downstream software module, so no additional manual cropping is required for prospective use.”

**3. Are images from the proprietary dataset publicly available?**

**Response:** Thank you for this question. The WBC images from the hospital cohort are derived from routine patient specimens and are currently not publicly available due to institutional data governance and patient privacy regulations. However, we are in discussion with our clinical collaborators about the possibility of releasing a de-identified subset in the future, subject to ethics and institutional approvals. In the meantime the public WBC dataset can be found at:

(a) PBC: <https://data.mendeley.com/datasets/snkd93bnjr/1>
(b) Raabin: https://raabindata.com/free-data/

We have included a section on data availability:

“The hospital WBC image dataset from Tan Tock Seng Hospital contains potentially sensitive patient information and cannot be shared publicly due to institutional data protection policies. Researchers who meet the criteria for access to this de-identified dataset may contact the NHG Research Data Oversight Committee Secretariat at nhggroup.RDOCSecretariat@nhghealth.com.sg.”
