# Supplementary material for: Aligning deep learning and interpretable models for blood cell classification: A dual-model framework for explainability
Source: PLOS Digit Health. 2026 Jul 30;5(7):e0001514. doi: 10.1371/journal.pdig.0001514 (PMC13422869; doi:10.1371/journal.pdig.0001514)
Supplement: Attachment — Submitted filename: Response to Reviewers_Rev2.docx [file pdig.0001514.s010.docx]

# **Reviewer Comments**

## **Response to the Academic Editor**

We thank the Academic Editor for the careful handling of our manuscript and for the opportunity to revise it. We have carefully addressed the comments raised during the review process and have revised the manuscript accordingly. A detailed point-by-point response is provided below.

## **Reviewer 1**

**1) Table 4: RF-GT results**

**Are these results for the RF model trained on the ground truth labels, or are they for the RF model trained on YOLO predictions but tested on the ground truth labels?**

**Response:** We thank the reviewer for this important clarification. The results reported as RF-GT correspond to a Random Forest model trained and evaluated using the ground-truth human labels. In contrast, RF-Y11 denotes the Random Forest model trained and evaluated using the YOLO11 predictions. In the DREAM framework, the explanation model is RF-Y11, because its purpose is to act as a feature-level surrogate of the YOLO classifier and to provide concept-based explanations for YOLO’s decisions.

**2) Methodology - Segmentation and feature generation**

**In the newly added paragraph, the authors state that they retain only cells with reasonable segmentation quality when training the explanation model. Could the authors clarify whether they have the ground truth segmentation masks for the proprietary dataset? If ground truth masks are not available, how does the authors assess segmentation quality in the proprietary dataset during cell selection?**

**Response:** We thank the reviewer for this important question. We do not have ground-truth segmentation masks for the proprietary dataset. In our study, the proprietary dataset provides expert annotations for classification labels, whereas quantitative segmentation evaluation is performed on the Mendeley public dataset, which includes expert-annotated nucleus and cytoplasm masks. Accordingly, the reported IoU and Dice values are obtained only from the Mendeley dataset. On that dataset, the proposed KMeans–SAM2 pipeline achieved mean IoU values above 0.85 and mean Dice values above 0.91, indicating good agreement with expert masks.

To further examine robustness, we analysed segmentation behaviour across the three image groups in the Mendeley dataset, which differ in colour characteristics. As shown in Fig. 6, all three groups exhibited slight cytoplasm over-segmentation, but ANOVA showed no statistically significant difference among groups. This suggests that the segmentation pipeline is reasonably robust to colour variation arising from staining and imaging differences. In addition, the consistent downstream performance observed across the proprietary dataset and the two public classification datasets further supports the likely transferability of the overall segmentation–feature extraction pipeline across datasets with differing image characteristics. We have therefore revised the manuscript to present this point as supporting evidence of robustness, rather than as direct proof of equivalent segmentation accuracy on the proprietary dataset.

For the proprietary dataset specifically, segmentation quality during cell selection is not assessed by IoU/Dice-based comparison, because no ground-truth masks are available. Instead, after segmentation and feature extraction, we apply a post-segmentation quality-control step by retaining only cells whose computed morphological measurements fall within a cell-type-specific plausible range, implemented as a mean ± 1 standard deviation guardrail on cell area. This filtering step is intended to reduce the influence of atypical or unstable segmentations before training the explanation model. We will revise the manuscript to make this distinction explicit, so that IoU/Dice-based evaluation is clearly described as applying only to the Mendeley dataset, whereas cell selection in the proprietary dataset is based on post-segmentation feature plausibility filtering rather than ground-truth mask comparison.

**Fig 6. Segmentation evaluation results on the cytoplasm.** Groups A, B and C showed distinct colour characteristics. All exhibited slight cytoplasm over-segmentation. ANOVA showed no statistically significant difference among groups.

The manuscript has been edited as follows:

“The challenge in cell segmentation lies primarily in detecting the interface between cytoplasm and background. The Mendeley dataset consists of three image groups with distinct colour characteristics. We therefore compared segmentation performance across Groups A, B, and C by analysing the area difference between the segmented cytoplasm and the corresponding ground-truth cytoplasm mask. Fig. 6 shows representative examples and the distribution of cytoplasm area differences. All three groups showed slight over-segmentation, and ANOVA indicated no significant difference among them. These results suggest that the segmentation method is reasonably robust to colour variation due to staining and imaging conditions. As ground-truth segmentation masks are unavailable for the proprietary dataset, IoU/Dice-based evaluation is performed only on the Mendeley dataset. To ensure reliable feature extraction, for training the explanation model on the proprietary dataset, we instead apply a post-segmentation plausibility filter by retaining only cells whose computed cell area falls within a cell-type-specific mean ± 1 standard deviation range.”

**3) Have the authors checked the SHAP results and RF residuals for cells that are correctly classified by YOLO, but wrongly classified by RF, or vice versa?Response: Response:** We thank the reviewer for this helpful suggestion. Yes, we have now extended the analysis to explicitly examine the four relevant scenarios:

(i) YOLO is correct and RF agrees with YOLO;

(ii) YOLO is correct but RF disagrees with YOLO;

(iii) YOLO is incorrect but RF agrees with YOLO; and

(iv) both YOLO and RF are incorrect.

In the fully concordant case, where YOLO agrees with the ground truth and RF agrees with YOLO, the occurrence rate is 98.86% and the mean RF residual is only 0.0424. In contrast, all three non-ideal scenarios are rare collectively (1.14%) and show substantially higher residuals. This indicates that disagreement involving either model is associated with a marked increase in residual error, supporting the interpretation of the residual as a useful indicator of weak surrogate support and elevated prediction risk.

We also revised the figure on SHAP analysis accordingly. In the fully concordant scenario, the dominant features for the test sample (S4 Fig) are consistent with the global class-level SHAP profile (S5 Fig). By contrast, in the discordant scenarios, the feature patterns show weaker overlap with the expected class-level profile, indicating that the explanation is less stable and less well aligned with the expected morphological evidence. Taken together, these results show that SHAP consistency and RF residual provide complementary information: SHAP reflects whether the predicted class is supported by the expected feature pattern, while the residual quantifies how closely the feature-level surrogate supports the YOLO decision.

We have revised both S1 Table and S4 Fig to include these additional cases, and we have updated the main text to make clear that the residual is lowest when both models are correct and increases substantially whenever disagreement or error occurs. This revision directly addresses the reviewer’s question regarding the “YOLO-correct/RF-wrong” and “YOLO-wrong/RF-correct” cases.

**S1 Table Comparison of Random Forest (RF) residuals based on YOLO predictions.** In the ideal scenario where YOLO predicted correctly and RF prediction aligns with YOLO prediction, mean RF residual is significantly lower than the suboptimal scenarios. In the evaluation, the ideal scenario has a good occurrence rate of 98.85%.

We have updated the manuscript as follows:

“To a large extent, the strongly activated areas coincide with the top attributes identified by SHAP analysis. We further examined four scenarios defined by YOLO correctness with respect to ground truth and RF agreement with YOLO: (i) both YOLO and RF are correct, (ii) YOLO is correct but RF disagrees, (iii) YOLO is incorrect but RF agrees, and (iv) both models are incorrect. In the fully concordant scenario, the SHAP profile of the test sample is consistent with the global class-level SHAP profile, with a mean RF residual of 0.0424 and an occurrence rate of 98.86%. In the remaining scenarios, which collectively account for 1.14% of cases, the mean RF residual is substantially higher and the SHAP patterns are less consistent with the expected class-level profile. These findings indicate that large residuals are associated with weaker surrogate support for the YOLO decision and with a higher likelihood of disagreement or error. This further supports the suitability of the engineered features for providing faithful feature-level explanations of the model predictions.”

**S4 Fig. SHAP analysis of test samples with incorrect and correct YOLO predictions.** Four scenarios are presented: (i) both YOLO and RF are correct, (ii) YOLO is correct but RF disagrees, (iii) YOLO is incorrect but RF agrees, and (iv) both models are incorrect. In the optimal scenario with full agreement, top features on the SHAP analysis of the sample align with the global SHAP profile (S5. Fig), whereas, in the other scenarios, the features overlap is weaker and the existence of a strong signal from unrelated feature tips the prediction over, leading to wrong predictions.

**S5 Fig. Global class-level SHAP profile.**

## **Reviewer 2**

**1. Surrogate Model Explains YOLO (excellently addressed)**

**The authors added S4 Fig, S5 Fig, and S1 Table demonstrating when RF explanations are reliable:**

**Mean ε: 0.0493 (YOLO correct) vs 0.6222 (YOLO incorrect) — 12.6× difference**

**YOLO–RF agreement: 99.02% vs 33.33%**

**Large ε effectively flags potential errors. The reframing from “RF explains YOLO” to “RF as a concept-level surrogate” is scientifically appropriate and addresses the original concern.**

**Minor suggestion (optional): Provide a recommended clinical threshold for ε.**

**Response:** We thank the reviewer for the positive assessment of this revision and for the helpful suggestion to provide a threshold for the residual ε. In response, we have now made this point explicit and expanded the analysis to better characterize when the RF surrogate is reliable. Specifically, we revised S1 Table to separate four scenarios: (i) both YOLO and RF are correct, (ii) YOLO is correct but RF disagrees, (iii) YOLO is incorrect but RF agrees, and (iv) both models are incorrect. This extended breakdown shows that in the fully concordant scenario, the mean RF residual is very low (0.0424) and the occurrence rate is high (98.86%), whereas in all other scenarios the residual is substantially higher, with a collective occurrence rate of only 1.14%. These results further support that large residuals are associated with weak surrogate support for the YOLO decision and with an increased likelihood of prediction error.

To provide a practical decision rule, we additionally analysed the distribution of RF residuals in the full-agreement subset and found it to be right-skewed rather than approximately Gaussian. We therefore derived a threshold using the Interquartile Range (IQR) rule $\left( {75}^{\text{th}}\text{ percentile}+1.5\times\mathrm{IQR} \right)$, which yielded a value of 0.1. As illustrated in S6 Fig, this threshold cleanly separates the high-confidence full-agreement cluster from the disagreement scenarios and can therefore serve as a practical operating threshold for identifying predictions that warrant closer review.

**S1 Table Comparison of Random Forest (RF) residuals based on YOLO predictions.** In the ideal scenario where YOLO predicted correctly and RF prediction aligns with YOLO prediction, mean RF residual is significantly lower than the suboptimal scenarios. In the evaluation, the ideal scenario has a good occurrence rate of 98.85%.

**S6 Fig. Distribution plot and box plot of RF residuals.** The distribution of full agreement subset (YOLO=GT, RF = YOLO) is significantly right-skewed. To account for this non-Gaussian behaviour, a statistical acceptance threshold was computed using the Interquartile Range (IQR) method (75^th^ percentile + 1.5 x IQR), arriving at 0.1. With this acceptance threshold, the framework establishes a conservative safety margin that effectively isolates the high-confidence full agreement prediction cluster from all scenarios of model disagreement.

We have also updated the manuscript accordingly:

“To a large extent, the strongly activated areas coincide with the top attributes identified by SHAP analysis. We further examined four scenarios defined by YOLO correctness with respect to ground truth and RF agreement with YOLO: (i) both YOLO and RF are correct, (ii) YOLO is correct but RF disagrees, (iii) YOLO is incorrect but RF agrees, and (iv) both models are incorrect. In the fully concordant scenario, the SHAP profile of the test sample is consistent with the global class-level SHAP profile (S4 and S5 Figs in Supporting information section), with a mean RF residual of 0.0424 and an occurrence rate of 98.86%. In the remaining scenarios, which collectively account for 1.14% of cases, the mean RF residual is substantially higher. These findings indicate that large residuals are associated with weaker surrogate support for the YOLO decision and with a higher likelihood of disagreement or error. Based on the residual distribution of the full-agreement subset, we further derived a practical operating threshold of 0.1 using the Interquartile Range (IQR) method $\left( {75}^{\text{th}}\text{ percentile}+1.5\times\mathrm{IQR} \right)$ (S6 Fig in Supporting information section). Predictions with residuals above this value may therefore warrant closer review. ”

**2. Segmentation Validation (substantially addressed)**

**Validation on the Mendeley dataset (400 cells, IoU >85%, Dice >91%) exceeds the originally requested scope. Quality filtering (1 SD threshold) and discussion of pale cytoplasm challenges demonstrate rigor and transparency.**

**Minor clarifications (optional): Report the filtering exclusion rate, provide per-cell-type breakdown, and briefly describe dataset characteristics.**

**Response:** We thank the reviewer for the positive assessment and for these helpful suggestions. We have revised the manuscript to clarify both the dataset characteristics and the role of quality filtering. Briefly, the proprietary dataset contains 23,227 Wright’s-stained single-cell images, while the public classification datasets differ in staining protocol, image size, and available cell types. Quantitative segmentation validation is performed on the Mendeley dataset, which contains 400 images with expert-annotated nucleus and cytoplasm masks.

For the proprietary dataset, where ground-truth segmentation masks are unavailable, the quality-control step is implemented as a cell-type-specific post-segmentation plausibility filter based on computed cell area. Specifically, for each cell type, we retain cells whose segmented area falls within a mean ± 1 standard deviation range, thereby reducing the influence of atypical or unstable segmentations before training the explanation model. We chose cell area for this guardrail because boundary uncertainty between cytoplasm and background is the main source of segmentation variability, making area the most directly affected and informative feature for filtering.

We have edited the manuscript as follows:

“To ensure reliable feature extraction, for training the explanation model on the proprietary dataset, we instead apply a post-segmentation plausibility filter by retaining only cells whose computed cell area falls within a cell-type-specific mean ± 1 standard deviation range. ”

**3–5. Scope and Limitations (adequately addressed)**

**The revised manuscript includes a comprehensive limitations section covering feature scope, pathological cells, and user study constraints. The discussion is appropriate and transparent, though explicitly mentioning advanced morphological features (e.g., chromatin structure, nucleoli) could further strengthen clarity.**

**Response:** We thank the reviewer for this helpful suggestion. We agree that explicitly stating the fine-grained morphological features not yet captured by the current framework improves the clarity of the limitations discussion. We have therefore revised the manuscript to clarify that, although the current engineered feature set captures major morphological properties such as size, circularity, colour, granularity, and lobe count, it does not yet fully represent more subtle diagnostic cues used in haematology practice, including chromatin structure, nucleoli, and lineage-specific inclusions such as Auer rods. We have added the following text:

“Future work will potentially use advanced generative augmentation such as diffusion models or GANs to mitigate class imbalance. We will also extend the evaluation to rarer and morphologically atypical cells, such as myeloblasts and lymphoblasts, including cases that require recognition of subtle diagnostic cues such as chromatin structure, nucleoli, and lineage-specific inclusions such as Auer rods. Additionally, while the current engineered features capture major morphological properties, they do not yet fully represent these finer-grained attributes. We therefore plan to investigate richer feature representations, together with improved segmentation robustness in low-contrast cytoplasmic regions and pauci-stained boundaries.”

**6. THOUGHTFUL LIMITATION**

**A general limitation of post-hoc XAI methods should be acknowledged: high faithfulness (RF approximates YOLO well) and plausibility (expert agreement) do not guarantee medically sound reasoning, as models may still rely on spurious correlations.**

**For example, if YOLO learned dataset-specific shortcuts (e.g., cell size correlating with class), the surrogate would faithfully capture this pattern while remaining clinically suboptimal under distribution shift.**

**The authors partially mitigate this risk through multi-dataset validation, spatial alignment (Score-CAM/SHAP), and error analysis showing RF–YOLO disagreement when YOLO fails. However, a brief acknowledgment of this faithfulness–validity gap in the Discussion would strengthen the manuscript.**

**Specifically, I suggest adding 1–2 sentences noting that high surrogate agreement reflects model faithfulness rather than guaranteed clinical correctness, and that current validation provides reasonable but not definitive assurance.**

**Response:** We thank the reviewer for this important suggestion. We agree that high agreement between the RF surrogate and YOLO reflects model faithfulness, but does not by itself guarantee clinical correctness. We have therefore revised the Discussion to clarify that, although our multi-dataset validation, spatial alignment, and residual-based error analysis provide supportive evidence, they do not fully exclude the possibility that the model may rely partly on dataset-specific shortcuts:

“A limitation of this approach is that high agreement between the RF surrogate and YOLO reflects model faithfulness rather than guaranteed clinical correctness. Although our cross-dataset evaluation, spatial alignment, and residual-based error analysis provide supportive evidence, they do not fully exclude the possibility that the model may rely partly on dataset-specific shortcuts or clinically suboptimal cues.”

**OPTIONAL SUGGESTIONS (NOT REQUIRED)**

**Minor improvements could include clearer S1 Table labeling, reporting segmentation filtering rates, providing per-class metrics in confusion matrices, and explicitly mentioning advanced morphological features.**

**Response:** We thank the reviewer for these helpful optional suggestions. Where appropriate, we have incorporated them in the revision. Specifically, S1 Table has been relabelled and expanded to distinguish the additional YOLO–RF agreement scenarios, and the manuscript now clarifies that segmentation filtering is implemented as a cell-type-specific mean ± 1 standard deviation plausibility filter on cell area. We have also revised the limitations discussion to explicitly mention finer-grained morphological cues as directions for future work. Per-class precision and recall are already reported in Table 4, while the confusion matrices are retained to visualize class-specific error patterns.

## **Reviewer 3**

**The authors have addressed all of my remarks.**

We sincerely thank Reviewer 3 for the careful evaluation of our revised manuscript and for noting that the concerns have been adequately addressed. We greatly appreciate the reviewer’s time and constructive feedback, which helped improve the paper.
