# Supplementary material for: Aligning deep learning and interpretable models for blood cell classification: A dual-model framework for explainability
Source: PLOS Digit Health. 2026 Jul 30;5(7):e0001514. doi: 10.1371/journal.pdig.0001514 (PMC13422869; doi:10.1371/journal.pdig.0001514)
Supplement: Peer review history

# Peer review: Decision Letter 0

**DOI:** 10.1371/journal.pdig.0001514.r001

**Authors:** Jian-Qing Zheng, Mahdi Ghiasi

18 Dec 2025

PDIG-D-25-00900

Aligning deep learning and interpretable models for blood cell classification: A dual-model framework for explainability

PLOS Digital Health

Dear Dr. Fan,

Thank you for submitting your manuscript to PLOS Digital Health. After careful consideration, we feel that it has merit but does not fully meet PLOS Digital Health's publication criteria as it currently stands. Therefore, we invite you to submit a revised version of the manuscript that addresses the points raised during the review process.

Please submit your revised manuscript by Feb 16 2026 11:59PM. If you will need more time than this to complete your revisions, please reply to this message or contact the journal office at digitalhealth@plos.org. When you're ready to submit your revision, log on to https://www.editorialmanager.com/pdig/ and select the 'Submissions Needing Revision' folder to locate your manuscript file.

Please include the following items when submitting your revised manuscript:

* A letter that responds to each point raised by the editor and reviewer(s). You should upload this letter as a separate file labeled 'Response to Reviewers'. This file does not need to include responses to any formatting updates and technical items listed in the 'Journal Requirements' section below.

* A marked-up copy of your manuscript that highlights changes made to the original version. You should upload this as a separate file labeled 'Revised Manuscript with Track Changes'.

* An unmarked version of your revised paper without tracked changes. You should upload this as a separate file labeled 'Manuscript'.

If you would like to make changes to your financial disclosure, competing interests statement, or data availability statement, please make these updates within the submission form at the time of resubmission. Guidelines for resubmitting your figure files are available below the reviewer comments at the end of this letter.

We look forward to receiving your revised manuscript.

Kind regards,

Jian-Qing Zheng

Guest Editor

PLOS Digital Health

Mahdi Ghiasi

Section Editor

PLOS Digital Health

Leo Anthony Celi

Editor-in-Chief

PLOS Digital Health

orcid.org/0000-0001-6712-6626

**Journal Requirements:**

1. Please amend your detailed Financial Disclosure statement. This is published with the article. It must therefore be completed in full sentences and contain the exact wording you wish to be published.

i. Please clarify all sources of financial support for your study. List the grants, grant numbers, and organizations that funded your study, including funding received from your institution. Please note that suppliers of material support, including research materials, should be recognized in the Acknowledgements section rather than in the Financial Disclosure.

ii. State the initials, alongside each funding source, of each author to receive each grant. For example: "This work was supported by the National Institutes of Health (####### to AM; ###### to CJ) and the National Science Foundation (###### to AM)."

iii. State what role the funders took in the study. If the funders had no role in your study, please state: “The funders had no role in study design, data collection and analysis, decision to publish, or preparation of the manuscript.”

iv. If any authors received a salary from any of your funders, please state which authors and which funders.

2. We ask that a manuscript source file is provided at Revision. Please upload your manuscript file as a .doc, .docx, .rtf or .tex.

3. We have noticed that you have uploaded Supporting Information files, but you have not included a list of legends. Please add a full list of legends for your Supporting Information files after the references list.

4. We note that you have included your Figures and Supporting Information within the body of your manuscript. Please remove the Figures and Supporting Information from the body of your manuscript as they are already uploaded in file inventory.

5. We note that your Data Availability Statement is currently as follows:

“The blood film images used for the development were provided by Tan Tock Seng Hospital in Singapore. Tan Tock Seng Hospital is a collaborator in this project. The blood film images belong to the hospital patients. Due to data protection, these images are not publicly available. To test that the work is replicable, the project is validated with 2 public datasets:

1) A dataset for microscopic peripheral blood cell images for development of automatic recognition systems. Authors: Acevedo, Andrea, Merino, Anna, Alférez, Santiago, Molina, Ángel, Boldú, Laura, Rodellar, José.

2) A large dataset of white blood cells containing cell locations and types, along with segmented nuclei and cytoplasm. Authors: Kouzehkanan, Zahra Mousavi, Saghari, Sepehr, Tavakoli, Sajad, Rostami, Peyman, Abaszadeh, Mohammadjavad, Mirzadeh, Farzaneh, Satlsar, Esmaeil Shahabi, Gheidishahran, Maryam, Gorgi, Fatemeh, Mohammadi, Saeed, others.”

Please confirm at this time whether or not your submission contains all raw data required to replicate the results of your study. Authors must share the “minimal data set” for their submission. PLOS defines the minimal data set to consist of the data required to replicate all study findings reported in the article, as well as related metadata and methods (https://journals.plos.org/plosone/s/data-availability#loc-minimal-data-set-definition).

For example, authors should submit the following data:

- The values behind the means, standard deviations and other measures reported;

- The values used to build graphs;

- The points extracted from images for analysis.

Authors do not need to submit their entire data set if only a portion of the data was used in the reported study.

If your submission does not contain these data, please either upload them as Supporting Information files or deposit them to a stable, public repository and provide us with the relevant URLs, DOIs, or accession numbers. For a list of recommended repositories, please see https://journals.plos.org/plosone/s/recommended-repositories.

If there are ethical or legal restrictions on sharing a de-identified data set, please explain them in detail (e.g., data contain potentially sensitive information, data are owned by a third-party organization, etc.) and who has imposed them (e.g., an ethics committee). Please also provide contact information for a data access committee, ethics committee, or other institutional body to which data requests may be sent. If data are owned by a third party, please indicate how others may request data access.

If the reviewer comments include a recommendation to cite specific previously published works, please review and evaluate these publications to determine whether they are relevant and should be cited. There is no requirement to cite these works unless the editor has indicated otherwise.

**Additional Editor Comments (if provided):**

The work addresses an important clinical need for interpretable AI in hematology. While the technical execution demonstrates competence and the classification performance is strong, the central claim that the RF model 'explains' YOLO predictions requires significant revision.

All three reviewers have provided valid and constructive feedback. Reviewer 1's fundamental methodological concern is particularly important. I recommend Major Revision with the understanding that the authors should either (1) provide rigorous quantitative evidence that RF truly explains YOLO's reasoning, or (2) reframe their claims more modestly while still highlighting the framework's genuine value as a dual-classification system with interpretable outputs.

[Note: HTML markup is below. Please do not edit.]

**Reviewers' Comments:**

Reviewer's Responses to Questions

**Comments to the Author**

1. Does this manuscript meet PLOS Digital Health’s publication criteria? Is the manuscript technically sound, and do the data support the conclusions? The manuscript must describe methodologically and ethically rigorous research with conclusions that are appropriately drawn based on the data presented.

Reviewer #1: Partly

Reviewer #2: Partly

Reviewer #3: Yes

**********

2. Has the statistical analysis been performed appropriately and rigorously?

Reviewer #1: Yes

Reviewer #2: Yes

Reviewer #3: Yes

**********

3. Have the authors made all data underlying the findings in their manuscript fully available (please refer to the Data Availability Statement at the start of the manuscript PDF file)?

The PLOS Data policy requires authors to make all data underlying the findings described in their manuscript fully available without restriction, with rare exception. The data should be provided as part of the manuscript or its supporting information, or deposited to a public repository. For example, in addition to summary statistics, the data points behind means, medians and variance measures should be available. If there are restrictions on publicly sharing data—e.g. participant privacy or use of data from a third party—those must be specified.

Reviewer #1: No

Reviewer #2: Yes

Reviewer #3: No

**********

4. Is the manuscript presented in an intelligible fashion and written in standard English?

PLOS Digital Health does not copyedit accepted manuscripts, so the language in submitted articles must be clear, correct, and unambiguous. Any typographical or grammatical errors should be corrected at revision, so please note any specific errors here.

Reviewer #1: No

Reviewer #2: Yes

Reviewer #3: Yes

**********

5. Review Comments to the Author

Please use the space provided to explain your answers to the questions above. You may also include additional comments for the author, including concerns about dual publication, research ethics, or publication ethics. (Please upload your review as an attachment if it exceeds 20,000 characters)

Reviewer #1: This work proposes DREAM, an interpretable blood cell classification framework for blood smear examination, which uses YOLO to detect and classify cells, segment cells via K-means clustering and SAM 2, extracts clinically meaningful cell features, and then uses a Random Forest (RF) classifier to explain YOLO’s predictions. The YOLO model achieves good classification performance, while the RF explainer demonstrates good performance in replicating YOLO’s predictions. The authors claim that the results of the SHAP analysis of the explainer align with those of Score-CAM on YOLO, supporting the capacity of the RF explainer to provide interpretable explanations for YOLO.

The authors provide a good overview of past work on using explainable AI for blood cell classification. Standard explainability techniques, namely Score-CAM and SHAP, are employed for model explanations. The authors extended the SHAP formulation to include the discrepancy between the predictions of YOLO and the RF classifier, enabling the calculation of the RF model’s explanation for the YOLO model’s prediction. The adoption of unsupervised K-means clustering and SAM 2 for cell segmentation is appropriate and reduces the need for manual annotation, though more quantitative evaluation of the segmentation performance would be beneficial. The DREAM framework demonstrates excellent generalisability in the PBC and Raabin datasets. Model validation by clinicians was conducted, which provides a valuable quantitative assessment of the quality of the explanations generated by SHAP analysis.

However, the reviewer does not fully support the authors’ conclusion and assumptions. Despite achieving comparable accuracy to the YOLO classifier, the RF classifier utilises features that are not directly generated by YOLO. The morphological features are generated using segmentations obtained with K-means clustering and SAM 2, so they may no longer fully correlate with the features used by YOLO for prediction. Though the authors claim that the RF model’s SHAP analysis highlights regions and features that are also looked at by YOLO (shown by Score-CAM), there is no guarantee that the YOLO model and the RF model have similar decision-making mechanisms. Therefore, the reviewer does not think the statement that the YOLO model’s predictions are interpretable via the RF model is appropriate. Since the RF model also achieves good blood cell classification accuracy, the authors should further clarify their choice of using YOLO’s prediction instead of the RF model’s prediction for cell classification.

Please see below for detailed comments from the reviewer. Additionally, the authors could further polish the manuscript by adopting a more academic style of English.

Major comments

Introduction

1) Page 2, lines 9-10: It would be helpful if the authors could elaborate on the current challenges and limitations of manual blood smear examination, preferably with quantitative information. References should be provided.

2) Page 2, lines 38-29: Please give examples or provide further explanations. References should be provided.

Related work

3) Page 4, lines 100-102: Please explain how the features were trained. This sentence is confusing.

4) Page 4, lines 107-109: Please include references and describe past research that adopted ResNet and CNN for blood cell classification.

5) Page 4, lines 119-121: Since the authors state that explaining DL model predictions “proved” to be difficult, please provide some proof and references.

6) Table 1: In the “Method” column, it would be helpful to provide more details on the classical ML methods adopted.

There are many other studies on blood cell classifications using classical ML or DL methodologies. Here are some examples:

• Lamberti 2021 https://doi.org/10.1016/j.cmpbup.2021.100023

• Zou et al. 2024 https://doi.org/10.1016/j.bspc.2024.106820

• Sherpa et al. 2025 https://doi.org/10.1002/ima.70084

The authors also mentioned other studies on blood cell classification in the “Explainable AI” subsection. Could the authors explain why they include these specific studies in the table? It would be more informative to include the results of more studies that proposed explainable/interpretable DL methods.

7) Page 5, lines 134-136: CAMs do not necessarily adopt a red to blue colour scheme.

Methodology

8) Prediction module: It would be helpful to explain why YOLO is selected for prediction.

9) Segmentation module: Some details on prompt optimisation (lines 212-213) are preferred. Also, please provide more descriptions of the fixed positions and the random positions. A figure or table would be helpful.

Results

10) Classification model: Have the authors attempted other augmentation methods, such as small scaling, translation and shearing?

11) Segmentation and feature generation: Would it be possible to quantitatively assess the performance of the segmentation method by manually creating some ground truth segmentations and comparing the predictions with the ground truths?

Explanation evaluation

12) It is nice to see that the RF classifier’s explanations align with YOLO’s Score-CAM heatmaps. However, since the evaluation is qualitative and the morphological features are not directly generated by YOLO, the reviewer doubts whether the authors can confirm that the explainer successfully interprets YOLO’s prediction.

ROaR evaluation

13) Please elaborate on why this evaluation confirms that the explainer successfully interprets YOLO’s predictions.

Conclusion

14) Again, could the authors really confirm that the RF classifier explains the decision-making of the YOLO classifier?

15) Some discussion on the study’s limitations would be helpful.

Minor comments

Introduction

16) Page 2, lines 14-15: The nucleus is a cell organelle, so the authors should remove the “cell” before “boundaries”. Also, please change “classify” to the past tense.

17) Page 2, lines 22-29: Some references for the statements would be useful.

Related work

18) Page 3, lines 93-94: Could the authors provide examples of the features that were extracted on the nucleus? Would “from the nucleus” or “of the nucleus” be more appropriate?

19) Page 4, line 104: Please include the unabbreviated form of ANN.

20) Page 4, line 127: “Leveraged on ResNet” does not sound grammatically correct.

21) Page 5, lines 136-137: Please provide references for the techniques.

22) Page 5, line 158: There seems to be a grammar mistake.

Methodology

23) Feature generation module: More details on how the features were derived and calculated computationally would be useful.

Results

24) Table 4: It would be useful to have one or two more decimal places for the performance metrics.

25) Page 10, lines 300-302: Please provide a reference for the time required for manual blood film examination.

ROaR evaluation

26) It is interesting to see that the classification accuracy of lymphocytes remains high after removing the top five features. Some hypotheses on potential reasons would be beneficial.

Reviewer #2: This manuscript proposes DREAM, a dual-model framework combining YOLO for blood cell classification with a Random Forest surrogate trained on engineered morphological features to provide SHAP-based explanations. The approach aims to bridge deep learning performance with clinically meaningful interpretability. The evaluation includes multi-dataset testing, CAM–SHAP alignment, ROaR feature ablation, and a clinician user study.

Strengths:

1.Innovative integration of deep learning and a feature-based surrogate to achieve clinically interpretable outputs.

2.Strong YOLO performance across proprietary and public datasets.

3.Well-designed explainability pipeline (SHAP, Score-CAM, ROaR).

4.Meaningful engagement with clinical experts; explanations rated highly (96.9%).

5.Clear writing and solid methodological structure.

Major Weaknesses (Require Minor Revision):

1.Surrogate model explains YOLO, not the ground truth.

Since the Random Forest surrogate is trained on YOLO predictions, SHAP reflects YOLO’s internal decision logic rather than clinical ground truth. Please include a brief analysis of several YOLO misclassified cases, showing how SHAP behaves when YOLO is incorrect and discussing the meaning of the residual term ε (i.e., how often it becomes large and what that implies about explanation reliability).

2.No quantitative segmentation validation.

The segmentation module (K-means + SAM2) is essential for feature extraction but is evaluated only visually. Please provide minimal quantitative validation (e.g., IoU/Dice or inter-annotator agreement on a small subset), or explicitly acknowledge this as a limitation and discuss its possible impact on downstream features.

3.Limited morphological feature set.

Discuss how more advanced descriptors (chromatin clumping, nucleoli, vacuolation, toxic granules, blast morphology) could be incorporated in future work.

4.Generalizability to pathological cells unclear.

Acknowledge the limitation of using primarily normal WBCs and outline plans for extension to abnormal/pathological samples.

5.User study limitations.

Small cohort (n=9) and limited cases; clarify limitations and propose improved future study designs.

Minor Weaknesses

1.Provide key RF hyperparameters (e.g., number of trees, depth).

2.Including confusion matrices for YOLO and RF would improve transparency.

3.CAM–SHAP figure captions could be slightly expanded for clarity.

Recommendation

Minor Revision

The manuscript is strong and suitable for publication after addressing the points above.

Reviewer #3: In this manuscript, Yu Cheong et al describe the DREAM framework they constructed that accurately classifies WBCs from peripheral blood smear images. DREAM consists of the prediction module (YOLO object detection and classification model), segmentation module that crops nuclei and cytoplasm form individual cells, feature generation module that extracts pre-specified engineered features such as RGB channels and geometric/morphologic cell features, and finally, explanation module, that uses RF model with SHAP explanations of individual features. The model was compared to ground truth established by manual labeling of the images by hematology experts.

There are numerous publications on blood cell classification models achieving very good accuracy, however, the main value of the dual model approach described in this manuscript is interpretability of the deep learning model. YOLO predictions and SHAP feature analysis of the explanation model predictions aligned at the same morphologic regions, which is encouraging. In addition, authors performed a feature ablation analysis (ROaR technique) with successive engineered feature removal, testing the model's accuracy with each removed feature. This showed expected performance decline with each removed feature, confirming that the model captures and depends on clinically relevant cell characteristics.

The manuscript is well-written and easy to follow.

All proposed figures and tables are relevant and consistent with the content of the manuscript.

Remarks:

1. My main concern are small validation sets (9,730 and 2,670 of cell images), although it is encouraging that accuracy was retained despite different staining methods and image sizes in the validation sets. Did authors perform any color corrections or other image alterations on the validations sets?

2. In terms of clinical applicability of this model and using it as an AI assistant tool for hemato-pathologists, images of peripheral blood smears used prospectively would need to be cropped first? What tool do the authors propose to prepare/crop images of individual cells for analysis? How much additional time would this take?

3. Are images from the proprietary dataset publicly available?

**********

6. PLOS authors have the option to publish the peer review history of their article (what does this mean?). If published, this will include your full peer review and any attached files.

**Do you want your identity to be public for this peer review?**  If you choose “no”, your identity will remain anonymous but your review may still be made public.

For information about this choice, including consent withdrawal, please see our Privacy Policy.

Reviewer #1: No

Reviewer #2: No

Reviewer #3: No

**********

[NOTE: If reviewer comments were submitted as an attachment file, they will be attached to this email and accessible via the submission site. Please log into your account, locate the manuscript record, and check for the action link "View Attachments". If this link does not appear, there are no attachment files.]

**Figure resubmission:**

While revising your submission, we strongly recommend that you use PLOS’s NAAS tool (https://ngplosjournals.pagemajik.ai/artanalysis) to test your figure files. NAAS can convert your figure files to the TIFF file type and meet basic requirements (such as print size, resolution), or provide you with a report on issues that do not meet our requirements and that NAAS cannot fix.

After uploading your figures to PLOS’s NAAS tool - https://ngplosjournals.pagemajik.ai/artanalysis, NAAS will process the files provided and display the results in the "Uploaded Files" section of the page as the processing is complete. If the uploaded figures meet our requirements (or NAAS is able to fix the files to meet our requirements), the figure will be marked as "fixed" above. If NAAS is unable to fix the files, a red "failed" label will appear above. When NAAS has confirmed that the figure files meet our requirements, please download the file via the download option, and include these NAAS processed figure files when submitting your revised manuscript.

**Reproducibility:**

To enhance the reproducibility of your results, we recommend that authors of applicable studies deposit laboratory protocols in protocols.io, where a protocol can be assigned its own identifier (DOI) such that it can be cited independently in the future. Additionally, PLOS ONE offers an option to publish peer-reviewed clinical study protocols. Read more information on sharing protocols at https://plos.org/protocols?utm_medium=editorial-email&utm_source=authorletters&utm_campaign=protocols

# Author comment: Author response to Decision Letter 1

**DOI:** 10.1371/journal.pdig.0001514.r002

14 Feb 2026

Attachment Submitted filename: Response to Reviewers.docx

# Peer review: Decision Letter 1

**DOI:** 10.1371/journal.pdig.0001514.r003

**Authors:** Jian-Qing Zheng, Mahdi Ghiasi, Jian-Qing Zheng, Mahdi Ghiasi

25 Mar 2026

PDIG-D-25-00900R1

Aligning deep learning and interpretable models for blood cell classification: A dual-model framework for explainability

PLOS Digital Health

Dear Dr. Fan,

Thank you for submitting your manuscript to PLOS Digital Health. After careful consideration, we feel that it has merit but does not fully meet PLOS Digital Health's publication criteria as it currently stands. Therefore, we invite you to submit a revised version of the manuscript that addresses the points raised during the review process.

Please submit your revised manuscript by April 24, 2026. If you will need more time than this to complete your revisions, please reply to this message or contact the journal office at digitalhealth@plos.org. When you're ready to submit your revision, log on to https://www.editorialmanager.com/pdig/ and select the 'Submissions Needing Revision' folder to locate your manuscript file.

Please include the following items when submitting your revised manuscript:

* A letter that responds to each point raised by the editor and reviewer(s). You should upload this letter as a separate file labeled 'Response to Reviewers'. This file does not need to include responses to any formatting updates and technical items listed in the 'Journal Requirements' section below.

* A marked-up copy of your manuscript that highlights changes made to the original version. You should upload this as a separate file labeled 'Revised Manuscript with Track Changes'.

* An unmarked version of your revised paper without tracked changes. You should upload this as a separate file labeled 'Manuscript'.

If you would like to make changes to your financial disclosure, competing interests statement, or data availability statement, please make these updates within the submission form at the time of resubmission. Guidelines for resubmitting your figure files are available below the reviewer comments at the end of this letter.

We look forward to receiving your revised manuscript.

Kind regards,

Jian-Qing Zheng, Ph.D.

Guest Editor

PLOS Digital Health

Leo Anthony Celi

Editor-in-Chief

PLOS Digital Health

orcid.org/0000-0001-6712-6626

**Journal Requirements:**

If the reviewer comments include a recommendation to cite specific previously published works, please review and evaluate these publications to determine whether they are relevant and should be cited. There is no requirement to cite these works unless the editor has indicated otherwise.

**Additional Editor Comments (if provided):**

[Note: HTML markup is below. Please do not edit.]

**Reviewers' Comments:**

Reviewer's Responses to Questions

**Comments to the Author**

1. If the authors have adequately addressed your comments raised in a previous round of review and you feel that this manuscript is now acceptable for publication, you may indicate that here to bypass the “Comments to the Author” section, enter your conflict of interest statement in the “Confidential to Editor” section, and submit your "Accept" recommendation.

Reviewer #1: (No Response)

Reviewer #2: (No Response)

Reviewer #3: All comments have been addressed

**********

2. Does this manuscript meet PLOS Digital Health’s publication criteria? Is the manuscript technically sound, and do the data support the conclusions? The manuscript must describe methodologically and ethically rigorous research with conclusions that are appropriately drawn based on the data presented.

Reviewer #1: Yes

Reviewer #2: Yes

Reviewer #3: Yes

**********

3. Has the statistical analysis been performed appropriately and rigorously?

Reviewer #1: Yes

Reviewer #2: Yes

Reviewer #3: Yes

**********

4. Have the authors made all data underlying the findings in their manuscript fully available (please refer to the Data Availability Statement at the start of the manuscript PDF file)?

The PLOS Data policy requires authors to make all data underlying the findings described in their manuscript fully available without restriction, with rare exception. The data should be provided as part of the manuscript or its supporting information, or deposited to a public repository. For example, in addition to summary statistics, the data points behind means, medians and variance measures should be available. If there are restrictions on publicly sharing data—e.g. participant privacy or use of data from a third party—those must be specified.

Reviewer #1: No

Reviewer #2: Yes

Reviewer #3: Yes

**********

5. Is the manuscript presented in an intelligible fashion and written in standard English?

PLOS Digital Health does not copyedit accepted manuscripts, so the language in submitted articles must be clear, correct, and unambiguous. Any typographical or grammatical errors should be corrected at revision, so please note any specific errors here.

Reviewer #1: Yes

Reviewer #2: Yes

Reviewer #3: Yes

**********

6. Review Comments to the Author

Please use the space provided to explain your answers to the questions above. You may also include additional comments for the author, including concerns about dual publication, research ethics, or publication ethics. (Please upload your review as an attachment if it exceeds 20,000 characters)

Reviewer #1: Minor comments and questions

1) Table 4: RF-GT results

Are these results for the RF model trained on the ground truth labels, or are they for the RF model trained on YOLO predictions but tested on the ground truth labels?

2) Methodology - Segmentation and feature generation

In the newly added paragraph, the authors state that they retain only cells with reasonable segmentation quality when training the explanation model. Could the authors clarify whether they have the ground truth segmentation masks for the proprietary dataset? If ground truth masks are not available, how does the authors assess segmentation quality in the proprietary dataset during cell selection?

3) Have the authors checked the SHAP results and RF residuals for cells that are correctly classified by YOLO, but wrongly classified by RF, or vice versa?

Reviewer #2: FINAL REVIEW VERSION

OVERALL ASSESSMENT

I commend the authors on a comprehensive revision that addresses all major concerns. The manuscript has been substantially strengthened through rigorous error analysis, quantitative segmentation validation, and appropriate reframing of claims.

Recommendation: Accept with minor revision

MAJOR COMMENTS

1. Surrogate Model Explains YOLO (excellently addressed)

The authors added S4 Fig, S5 Fig, and S1 Table demonstrating when RF explanations are reliable:

Mean ε: 0.0493 (YOLO correct) vs 0.6222 (YOLO incorrect) — 12.6× difference

YOLO–RF agreement: 99.02% vs 33.33%

Large ε effectively flags potential errors

The reframing from “RF explains YOLO” to “RF as a concept-level surrogate” is scientifically appropriate and addresses the original concern.

Minor suggestion (optional): Provide a recommended clinical threshold for ε.

2. Segmentation Validation (substantially addressed)

Validation on the Mendeley dataset (400 cells, IoU >85%, Dice >91%) exceeds the originally requested scope. Quality filtering (1 SD threshold) and discussion of pale cytoplasm challenges demonstrate rigor and transparency.

Minor clarifications (optional): Report the filtering exclusion rate, provide per-cell-type breakdown, and briefly describe dataset characteristics.

3–5. Scope and Limitations (adequately addressed)

The revised manuscript includes a comprehensive limitations section covering feature scope, pathological cells, and user study constraints. The discussion is appropriate and transparent, though explicitly mentioning advanced morphological features (e.g., chromatin structure, nucleoli) could further strengthen clarity.

MINOR COMMENTS

RF hyperparameters (150 trees, depth 15), confusion matrices (Fig 4), and improved CAM–SHAP figure captions have all been appropriately added.

THOUGHTFUL LIMITATION

A general limitation of post-hoc XAI methods should be acknowledged: high faithfulness (RF approximates YOLO well) and plausibility (expert agreement) do not guarantee medically sound reasoning, as models may still rely on spurious correlations.

For example, if YOLO learned dataset-specific shortcuts (e.g., cell size correlating with class), the surrogate would faithfully capture this pattern while remaining clinically suboptimal under distribution shift.

The authors partially mitigate this risk through multi-dataset validation, spatial alignment (Score-CAM/SHAP), and error analysis showing RF–YOLO disagreement when YOLO fails. However, a brief acknowledgment of this faithfulness–validity gap in the Discussion would strengthen the manuscript.

Specifically, I suggest adding 1–2 sentences noting that high surrogate agreement reflects model faithfulness rather than guaranteed clinical correctness, and that current validation provides reasonable but not definitive assurance.

OPTIONAL SUGGESTIONS (NOT REQUIRED)

Minor improvements could include clearer S1 Table labeling, reporting segmentation filtering rates, providing per-class metrics in confusion matrices, and explicitly mentioning advanced morphological features.

Reviewer #3: The authors have addressed all of my remarks.

**********

7. PLOS authors have the option to publish the peer review history of their article (what does this mean?). If published, this will include your full peer review and any attached files.

**Do you want your identity to be public for this peer review?**  If you choose “no”, your identity will remain anonymous but your review may still be made public.

For information about this choice, including consent withdrawal, please see our Privacy Policy.

Reviewer #1: No

Reviewer #2: No

Reviewer #3: No

**********

[NOTE: If reviewer comments were submitted as an attachment file, they will be attached to this email and accessible via the submission site. Please log into your account, locate the manuscript record, and check for the action link "View Attachments". If this link does not appear, there are no attachment files.]

**Figure resubmission:**

While revising your submission, we strongly recommend that you use PLOS’s NAAS tool (https://ngplosjournals.pagemajik.ai/artanalysis) to test your figure files. NAAS can convert your figure files to the TIFF file type and meet basic requirements (such as print size, resolution), or provide you with a report on issues that do not meet our requirements and that NAAS cannot fix.

After uploading your figures to PLOS’s NAAS tool - https://ngplosjournals.pagemajik.ai/artanalysis, NAAS will process the files provided and display the results in the "Uploaded Files" section of the page as the processing is complete. If the uploaded figures meet our requirements (or NAAS is able to fix the files to meet our requirements), the figure will be marked as "fixed" above. If NAAS is unable to fix the files, a red "failed" label will appear above. When NAAS has confirmed that the figure files meet our requirements, please download the file via the download option, and include these NAAS processed figure files when submitting your revised manuscript.

**Reproducibility:**

To enhance the reproducibility of your results, we recommend that authors of applicable studies deposit laboratory protocols in protocols.io, where a protocol can be assigned its own identifier (DOI) such that it can be cited independently in the future. Additionally, PLOS ONE offers an option to publish peer-reviewed clinical study protocols. Read more information on sharing protocols at https://plos.org/protocols?utm_medium=editorial-email&utm_source=authorletters&utm_campaign=protocols

# Author comment: Author response to Decision Letter 2

**DOI:** 10.1371/journal.pdig.0001514.r004

22 Apr 2026

Attachment Submitted filename: Response to Reviewers_Rev2.docx

# Editor report: Decision Letter 2

**DOI:** 10.1371/journal.pdig.0001514.r005

**Authors:** Jian-Qing Zheng, Mahdi Ghiasi, Jian-Qing Zheng, Mahdi Ghiasi, Dhiya Al-Jumeily OBE, Jian-Qing Zheng

6 Jun 2026

Aligning deep learning and interpretable models for blood cell classification: A dual-model framework for explainability

PDIG-D-25-00900R2

Dear Dr Fan,

We are pleased to inform you that your manuscript 'Aligning deep learning and interpretable models for blood cell classification: A dual-model framework for explainability' has been provisionally accepted for publication in PLOS Digital Health.

Before your manuscript can be formally accepted you will need to complete some formatting changes, which you will receive in a follow-up email from a member of our team.

Please note that your manuscript will not be scheduled for publication until you have made the required changes, so a swift response is appreciated.

IMPORTANT: The editorial review process is now complete. PLOS will only permit corrections to spelling, formatting or significant scientific errors from this point onwards. Requests for major changes, or any which affect the scientific understanding of your work, will cause delays to the publication date of your manuscript.

If your institution or institutions have a press office, please notify them about your upcoming paper to help maximize its impact. If they'll be preparing press materials, please inform our press team as soon as possible -- no later than 48 hours after receiving the formal acceptance. Your manuscript will remain under strict press embargo until 2 pm Eastern Time on the date of publication. For more information, please contact digitalhealth@plos.org.

Thank you again for supporting Open Access publishing; we are looking forward to publishing your work in PLOS Digital Health.

Best regards,

Jian-Qing Zheng, Ph.D.

Guest Editor

PLOS Digital Health

***********************************************************

**Additional Editor Comments (if provided):**

**Reviewer Comments (if any, and for reference):**
